# Assessing the impact of phylogenetic reconstruction error on genetic reassortment inference in segmented viruses

**DOI:** 10.1371/journal.pone.0360852

**Published:** 2026-10-22

**Authors:** Hugo G. Castelán-Sánchez, Art F. Y. Poon

**Affiliations:** 1 Department of Pathology & Laboratory Medicine, Western University, London, Ontario, Canada; 2 Department of Microbiology & Immunology, Western University, London, Ontario, Canada; 3 Department of Computer Science, Western University, Rm. 355, Middlesex College, London, Canada; St Paul's Hospital Millennium Medical College, ETHIOPIA

## Abstract

Recombination is an important process in the evolution of RNA viruses. For viruses with segmented genomes, recombination can occur by reassortment of entire genomic segments. This process has played a significant role in the adaptation and spread of influenza viruses with novel genomic combinations, *i.e.*, antigenic shifts. Reassortment is usually identified by discrepancies between the topologies of trees reconstructed from different genomic segments, *e.g.*, subtree prune and regraft (SPR) rearrangements. However, this phylogenetic discordance can also result from errors in tree reconstruction due to insufficient information. The objective of this study was to evaluate the contribution of phylogenetic reconstruction error to the inference of reassortment events from influenza A virus (IAV) genomes from avian hosts. We curated a dataset of 11,795 complete avian H5Nx IAV genomes and sampled subsets of HA sequences with different expected levels of reconstruction error. Next, we simulated molecular evolution along the trees reconstructed from these HA samples, varying rates of evolution and sequence lengths to correspond to the other genomic segments. Because these simulations had no reassortment, any phylogenetic discordance identified from the resulting trees must be caused by reconstruction error. We found that simulations from random samples yielded about 32% as many SPRs as obtained from real data. This false positive rate was reduced to 4% when simulating trees from the most genetically divergent subset of HA sequences. By modifying the simulated trees with SPR rearrangements, we observed a false negative rate of 8.5%, which increased with the number of SPRs per tree. Overall, we found that reassortment can be substantially overestimated from phylogenetic discordance, underscoring the need to account for phylogenetic uncertainty and sampling design when analyzing IAV datasets for reassortment.

## Introduction

Recombination is an important mechanism of genomic evolution in RNA viruses [1]. This process involves the exchange of genetic material between different genomic backgrounds, facilitated by coinfection of a host with two or more distinct viral lineages. Recombination uncouples the evolutionary fate of a mutation from the rest of the genome, and under certain conditions [2], it will increase the diversity of genotypes in the population [3,4]. Many RNA viruses have segmented genomes, including members of the *Reoviridae* and *Orthomyxoviridae* families [5]. There are also many multipartite viruses that package their genomic segments into separate virus particles [6]. In segmented RNA viruses, recombination can occur by reassortment, in which the genomic segments packaged into a virion are derived from different parental genomes. This wholesale exchange of segments can accelerate the acquisition of combinations of mutations that are required to overcome host barriers more rapidly than the progressive accumulation of individual mutations [7].

Influenza A virus (IAV; family *Orthomyxoviridae*, genus *Alphainfluenza*) illustrates the potential impacts of genetic reassortment on the health of human and non-human animal populations. This virus has caused numerous severe epidemics and pandemics [8], several of which are associated with a reassortant virus [9].

IAV has a negative-sense RNA genome that consists of eight genomic segments that encode 10 major proteins and other accessory gene products [10].

When a host cell becomes co-infected with two different IAV genomes, there are 2^8^ = 256 possible combinations of genome segments that can be packaged into a new virus particle. Only two of these combinations are derived from the same parental genome. With the simplifying assumption of independent assortment of genome segments during packaging, the probability of packaging a reassortant genome is (256−2)/256≈99%. The co-circulation of multiple IAV subtypes and clades in domesticated animal host species provides abundant opportunities for co-infection and reassortment between divergent lineages [11,12]. Consequently, reassortment can generate novel chimeric IAV genomes, potentially enhancing the virus’s ability to transmit to new host species and establish productive infections [13,14].

The comparative analysis of IAV genome sequences plays an important role in measuring the frequency of reassortment and detecting the emergence of new reassortant viruses [15,16].

To this end, a number of different methods have been developed to identify reassortment events. In the absence of recombination, the evolutionary history of a sample population of genome sequences may be represented by a single phylogenetic tree. Reassortment uncouples the evolutionary histories of the affected genomic segments.

Consequently, a common technique to detect reassortment is to reconstruct a phylogeny for each segment and then to identify discrepancies in the topology and/or labels among the resulting trees.

Discordant trees are often visualized as tanglegrams [17], in which two trees sharing a common set of labels are drawn in opposite orientations, with subtrees rotated to maximize concordance of tip labels between the trees. Tanglegrams can be used to visually assess a small number of reassortment events. In addition, the optimization algorithm for untangling pairs of trees provides a rough measure of the extent of discordance that may be due to recombination, *i.e.*, entanglement. However, tanglegrams do not readily scale to the resolution of large numbers of reassortment events. Evolutionary events that produce discordant trees can be explicitly modeled using an ancestral recombination graph (ARG) [18]. Although there has been a recent resurgence of interest in ARGs [19], they remain too computationally complex for large-scale application.

A compromise between these two extremes is to infer the frequency of reassortment events by the number of tree rearrangement operations required to convert one segment tree to another [20], which can be interpreted as an edit distance. Screening for tips that transition between different labeled subtrees in the respective phylogenies is an equivalent approach [21,22]. There are many edit distances available for this purpose, including the Robinson-Foulds distance [23], tree bisection and reconnection [24], nearest neighbor interchange [25], and subtree prune and regraft [26]. Among these, the SPR distance is frequently used in studies of lateral gene transfer and recombination [27], possibly because the SPR most closely resembles the effect of recombination on the topologies of trees on either side of the breakpoint.

In this work, we performed a phylogenetic analysis starting from nearly 12,000 full-genome sequences of influenza A H5Nx viruses to identify putative rearrangement events from phylogenetic discordance. Our main objective is to evaluate the reliability of this approach, given the uncertainty in reconstructing accurate phylogenies under realistic conditions. To this end, we reconstructed phylogenies from samples of IAV H5Nx segment 4 sequences (encoding hemagglutinin, HA), and then used the resulting trees as the reference for simulating sequence evolution for the other segments. Because these sequences were simulated in the absence of reassortment, any inferred reassortments can be interpreted as false positives. Additionally, we modified the simulated trees with SPR rearrangements to measure false negative rates. We compared the entanglement values and SPR distances between the reference HA tree and the trees for all other segments in both real and simulated datasets to assess the specificity and sensitivity of these methods for detecting reassortment. Furthermore, we characterized the SPR rearrangements inferred from these data in terms of subtree size and the distance traversed in the tree, to determine whether certain types of SPRs are more likely to be genuine.

## Materials and methods

### Data collection

We retrieved sets of influenza A virus genome segments in which the segment 4 sequence encoding hemagglutinin (HA) was classified as subtype H5, and for which samples were isolated from avian hosts, from the NCBI GenBank and GISAID databases (accessed 5 May 2024). These genomes were retrieved irrespective of subtype classification for segment 6 encoding neuraminidase (N1–N9), pathogenicity classification (HPAI/LPAI), or clade assignment; hence, they are denoted as H5Nx. The GISAID query returned 5,994–6,000 sequences per segment, and the GenBank query returned 6,157–6,170 sequences per segment.

We combined these sequence data sets by genomic segment. Only a small proportion of sequences were exact duplicates across databases. For example, out of 12,167 neuraminidase (NA) sequences, there were 8,347 unique sequences, of which 1,417 (17%) had two or more instances — only 171 (12%) of these were represented in both databases, involving a total of 673 sequence records. This low amount of overlap is largely due to a transition from depositing sequences in GenBank to GISAID around 2020, coinciding with recent outbreaks of avian H5N1 IAV (S1 Fig). Sequence identity alone was insufficient to determine whether the sequences were derived from the same isolate. The dataset was not stratified by subtype, pathogenicity, host category, or geographic region, as our objective was to evaluate methodological sensitivity rather than lineage-specific epidemiological dynamics. Consequently, we merged these datasets by harmonizing the sequence labels into the World Health Organization [28] nomenclature format, including host species, country of sampling, isolate name, and year of sampling, and then filtering sequences with duplicate labels. This reduced the combined data to 11,795 sequences per segment. We generated alignments for each segment using MAFFT [29], v7.490;], removed duplicate sequences, and then manually inspected and cleaned each segment alignment with trimAl [30], v1.5.0].

We reconstructed a maximum likelihood phylogeny for the HA alignment using the standard generalized time-reversible (GTR) model with gamma-distributed rate variation and empirical base frequencies in IQ-TREE2 [31]. Next, we applied two subsampling strategies to reduce the number of sequences and modulate the amount of phylogenetic information. First, we progressively removed the shortest terminal branches from the HA phylogeny, using the prune function of the Biopython Phylo module, until a target tree length of 0.08 expected substitutions per nucleotide site was attained. This resulted in a subsample of the *n* = 213 most genetically divergent HA sequences.

In addition, we generated five independent random samples of *n* = 213 HA sequences to represent more typical levels of phylogenetic information. We used the labels of the sampled HA sequences to retrieve the corresponding sequences for the other seven segments and reconstruct trees. These two sampling strategies provided a means of evaluating the effect of phylogenetic reconstruction error on levels of phylogenetic discordance.

### Data simulation

To quantify the discordance among phylogenies reconstructed from sequences that evolved in the complete absence of reassortment, we simulated sequence alignments for each of the other segments along the tree inferred from the real HA alignment (*T*_HA_). Since every segment would evolve along the exact same tree topology, any reassortment event inferred from the simulated trees could be interpreted as a false positive. We proportionally rescaled branch lengths in *T*_HA_ so that the total tree length matched the length of the tree reconstructed from the actual sequences for that segment. Our motivation for adjusting tree lengths in this manner was to accommodate substantial variation in evolutionary rates among segments, which could otherwise confound our assessment of false-positive rates. We recognize that the evolutionary patterns of individual influenza A genome segments can vary markedly in natural populations, reflecting subtype-specific diversification, distinct reassortment histories, and deep lineage structuring (for example, among NA subtypes) [32]. However, our focus here was purely methodological: to measure the level of discordance that would arise under perfect topological congruence, thereby separating the impact of phylogenetic reconstruction error from that of genuine biological reassortment. For each rescaled tree, we used the Python module *Pyvolve* [33] to simulate sequence evolution under the default Goldman-Yang codon substitution model with the dN/dS ratio set to 0.5 and sequence length matched to each genomic segment.

### Phylogenetic analysis

We used two approaches to infer reassortment events. First, we used a tanglegram, a graphical comparison of two trees sharing a common set of tip labels. The trees are rotated and optionally re-rooted to minimize the discordance in the vertical location of labels, as quantified by the entanglement statistic. Our working assumption is that increasing amounts of reassortment will induce greater amounts of entanglement between trees from different segments. We used the *step2side* method, implemented in the Python module *tanglegram* [34], to disentangle the HA tree and the trees from every other segment. We further visualized the tanglegram using the cophylo function from the phytools package in R, which implements a closest-neighbor (NN) tanglegram approach by heuristically rotating the trees to minimize link crossings between matched tip labels [35]. Second, we used the *Espalier* library [36] in Python to efficiently calculate the approximate number of SPR (subtree prune and regraft) operations required to convert one phylogenetic tree into another. This number provides another means of estimating the number of reassortment events between phylogenetic trees.

We obtained SPR distances between the HA tree and the trees for every other segment for the actual IAV genome sequence data, and we made the same comparisons for trees reconstructed from simulated data.

We hypothesized that false positive reassortment events due to phylogenetic uncertainty would tend to involve SPR moves separated by shorter distances in the respective trees. To evaluate this hypothesis, we quantified the total path length separating the inferred graft points for each SPR operation.

Let the tree reconstructed from real sequences for the *i*-th segment be denoted by $T_{i}$. Moreover, let the tree reconstructed from simulated sequences that were evolved along the real HA tree *T*_4_ be denoted by $T_{i}^{*}$. For each subtree $S_{j}$ in the maximum agreement forest between trees *T*_4_ and $T_{i}$ for i≠4, we first located $S_{j}$ in the reference (segment 4) tree *T*_4_ by searching for the internal node with the fewest descendants that match all tip labels in $S_{j}$. We call this node the root of the subtree *j* in *T*_4_ and denote it as $r_{4}^{\left(j\right)}$. Next, we located the internal node in *T*_4_ with the same direct common ancestor as $r_{4}^{\left(j\right)}$, which we refer to as its sibling node, $s_{4}^{\left(j\right)}$. Switching to the other segment tree $T_{i}$, we located the same subtree $S_{j}$ to identify $r_{i}^{\left(j\right)}$ by matching tip labels. Finally, we located the internal node in $T_{i}$ with the fewest descendants that match all tip labels associated with $s_{4}^{\left(j\right)}$, which we denote as $s_{i}^{\left(j\right)}$. The sibling distance is then defined as the total path length (*i.e.*, sum of branch lengths) from $s_{i}^{\left(j\right)}$ to $r_{i}^{\left(j\right)}$. Put another way, $s_{i}^{\left(j\right)}$ is the approximate location from which the SPR operation pruned the subtree, and $r_{i}^{\left(j\right)}$ is the location to which the subtree was grafted. A diagram summarizing this procedure is provided as S2 Fig.

Following this method, we calculated the sibling distances for all SPR events between *T*_4_ and both real ($T_{i}$) and simulated ($T_{i}^{*}$) trees for other segments.

To measure the false negative rate (FNR), we simulated reassortment events on the HA tree with maximum divergence (*n* = 213). Known reassortment events were introduced by directly applying a fixed number of SPR rearrangements to the reference tree uniformly at random using a custom Python script. We simulated 100 replicates under two different scenarios: one with a single SPR event per replicate, and another with *10* independent SPR events per replicate. To record ‘ground truth’, we tracked the tip labels for all descendants of the donor branch associated with each SPR event.

Next, Espalier was used to build the maximum agreement forest (MAF) between the reference and reassortant trees for each replicate. For each MAF component, the descendant-tip set was compared with the donor branch in the previously recorded database. A reassortment event was only deemed to be successfully retrieved when all of the descendant taxa for a MAF were present in the reassortment event. When multiple SPR events occurred in the same replicate, each MAF component was matched one-to-one with a simulated donor branch, to ensure that each MAF was linked to a single donor branch. Event-level sensitivity (1-FNR) was defined as the percentage of donor branches that were fully recovered.

## Results

### Data collection

We obtained a total of 11,795 H5Nx influenza A virus genome sequences from the NCBI GenBank and GISAID databases. These sequences represented a variety of avian hosts, with the most frequent annotations being ‘chicken’ (*n* = 2,968), ‘duck’ (*n* = 1,774), and ‘turkey’ (*n* = 927). There was a total of 527 unique annotations for host organisms at varying taxonomic levels; for instance, mallard duck (*n* = 47) and Muscovy duck (*n* = 199) were both sub-classifications below ‘duck’. Similarly, there were 621 unique geolocation annotations, with the three most common being ‘Netherlands’ (*n* = 845), ‘Minnesota’ (*n* = 569), and ‘Vietnam’ (*n* = 557). Many records were labeled by state or province rather than by country, obscuring the relative abundance of samples from China and the United States. We reconstructed a maximum-likelihood phylogeny of all HA (segment 4) sequences, and then we generated a subsample of *n* = 213 (1.8%) sequences that maximized the total tree length for a given number of sequences by progressively removing the shortest terminal branches from this phylogeny. Next, we extracted the corresponding sequences for the other segments (*i.e.*, from the same virus isolates as the HA sample) and regenerated maximum likelihood phylogenies for each segment. These seven datasets are intended to minimize the error in phylogenetic reconstruction for a given sample size and will be referred to as the *control samples*. In addition, we generated five random samples with the same number of HA sequences, extracted sequences from the other segments, and reconstructed phylogenies using the same workflow. These random samples are meant to be more consistent with a conventional phylogenetic study.

### Tanglegram analysis

To visually assess the potential extent of reassortment in the evolutionary history of avian H5Nx genomes, we generated tanglegrams for the HA tree against the trees of every other segment. A tanglegram is a graphical method that re-roots and rotates branches in two trees with matching labels to minimize the discordance in the vertical positions of their labels.

Discordance can be quantified by an entanglement statistic that sums the differences in the vertical positions of each label and normalizes this total so that the statistic ranges from 0 (completely congruent) to 1 (worst-case scenario).

For example, the tanglegram between trees relating HA and NA segments from a random sample of genomes shows substantial entanglement (0.13; Fig 1A), implying that multiple reassortment events have occurred in the evolutionary history of these sequences.

**Fig 1:**
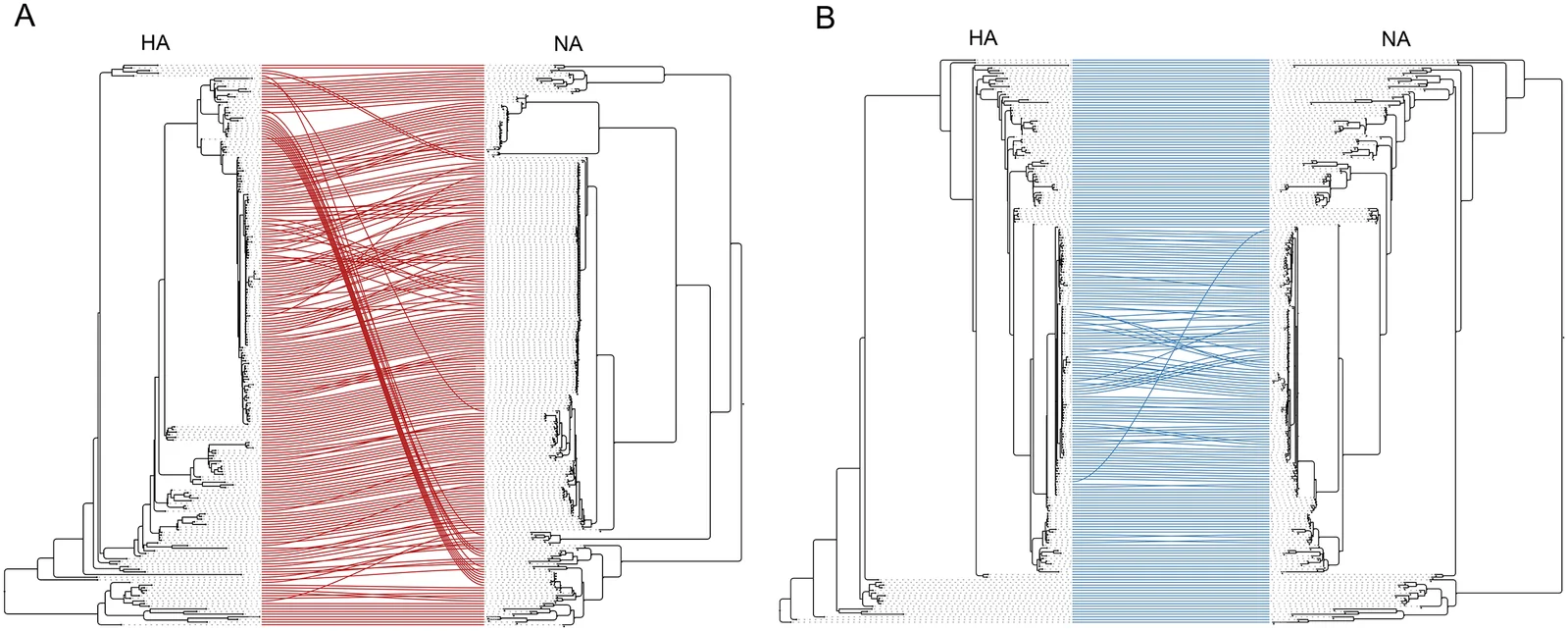
Tanglegrams between a random sample of *n* = 213 influenza A virus H5Nx sequences for segments 4 (HA) and 6 (NA). The NA tree (right side) was reconstructed either from actual segment 6 sequences from the same isolates (A), or generated by simulating sequence evolution along the rescaled HA tree. The entanglement values associated with these pairs of trees were 0.13 (actual) and 0.07 (simulated), respectively. An alternate version using the Python module *baltic* is provided as S3 Fig.

However, these results do not indicate how much discordance can be attributed to inaccuracies in reconstructing the respective phylogenies. To illustrate, Fig 1B displays a tanglegram between the same HA tree and a tree relating sequences simulated along the HA tree adjusted to the sequence length and molecular clock of the segment encoding NA.

Since the underlying phylogenies are congruent, these simulations provide an estimate of the expected amount of phylogenetic discordance in the absence of reassortment.

A multiple linear regression showed that simulated trees had significantly lower entanglement than trees reconstructed from real sequences (β = −0.12, t=−10.76, 95% CI: −0.14 to −0.10, $P<2\times 10^{-16}$, Fig 2).

**Fig 2:**
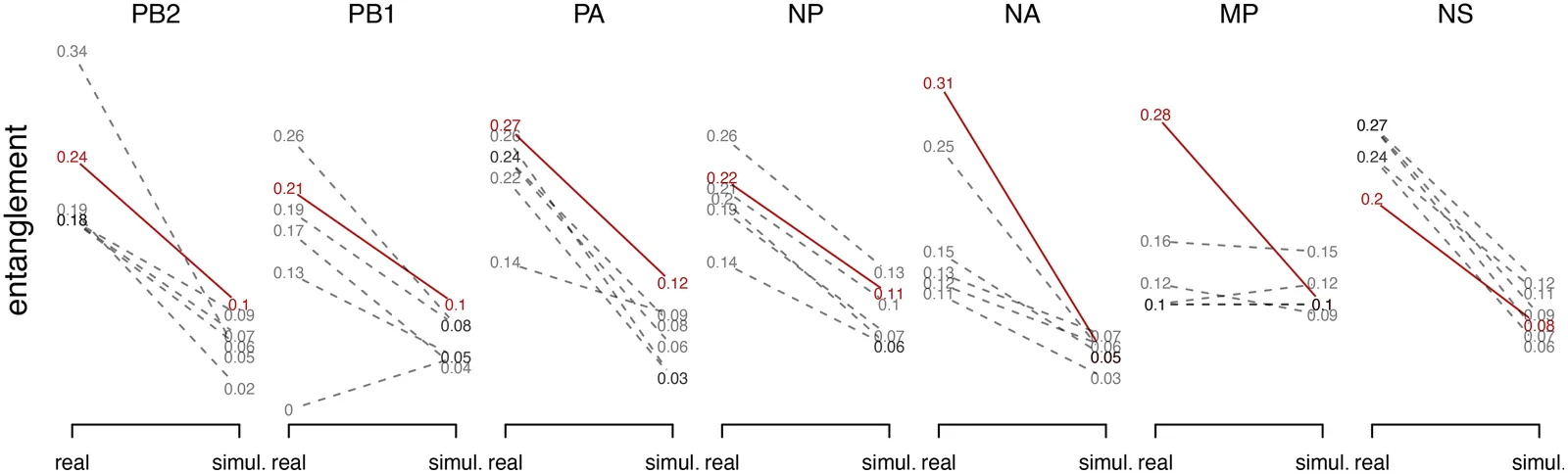
Slopegraphs summarizing entanglement values for real and simulated trees. Solid (red) lines indicate entanglement values obtained from the control (maximum-diversity) sample of HA sequences, and dashed lines indicate random samples. ‘Real’ values were obtained for trees reconstructed from actual sequences for the other segments. Simulated (‘simul.’) values were obtained for trees reconstructed from sequences simulated along the rescaled HA tree.

Put another way, a tree reconstructed from sequences simulated without reassortment was about 48% less ‘entangled’ than a tree from actual sequences. We also observed significantly lower entanglement in trees generated from random samples (β= −0.04, 95% CI: −0.07,−0.01, $P=8.5\times 10^{-3}$) relative to control samples. However, entanglement is not a reliable statistic for measuring phylogenetic discordance [37] and is difficult to interpret in terms of the extent of reassortment between segments.

### SPR analysis

A more robust and informative method for quantifying the discordance between phylogenies is the minimum number of subtree-prune-regraft (SPR) rearrangements required to transform one tree topology into another. This number defines an edit distance between the trees. A useful characteristic of SPR is that this rearrangement is similar to the effect of reassortment on trees [20].

We calculated the SPR distances between the same pairwise comparisons of trees as we used for entanglement. As expected, the effects of reassortment and phylogenetic reconstruction error were much clearer using SPR counts (Fig 3).

**Fig 3:**
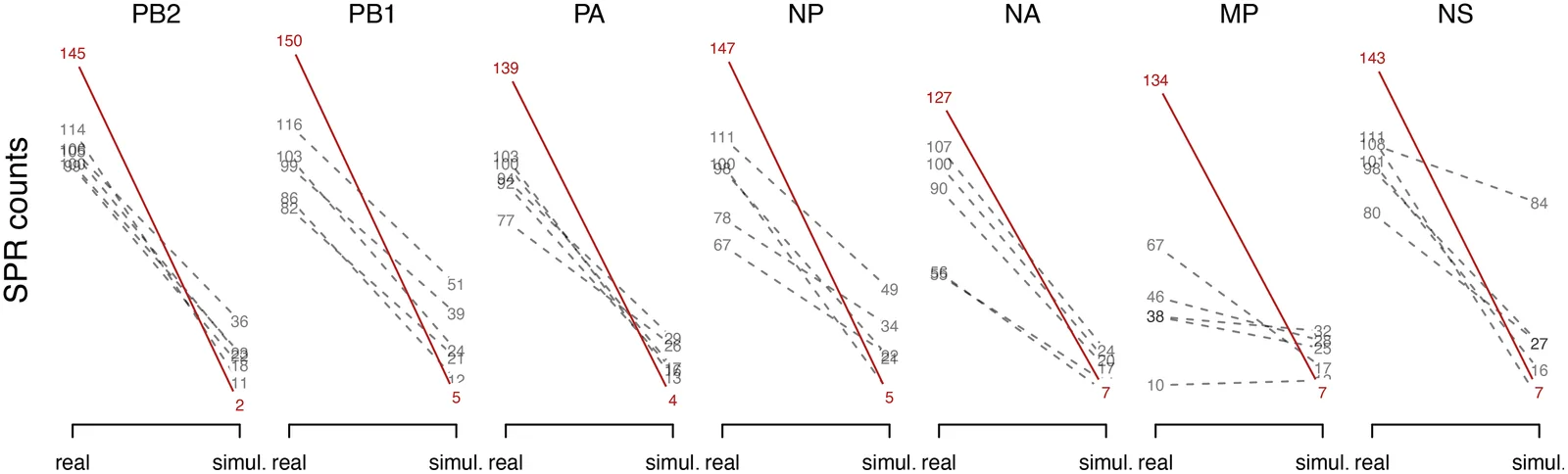
Slopegraphs summarizing subtree-prune regraft distances (minimum SPR counts) between real and simulated trees. Solid (red) lines indicate SPR counts obtained for the control sample of HA sequences, and dashed lines indicate random samples. ‘Real’ counts were obtained for trees reconstructed from actual sequences for other segments. Simulated (‘simul.’) counts were obtained for trees reconstructed from sequences simulated along the rescaled HA tree.

SPR counts were significantly lower in simulated trees without reassortment (negative binomial generalized linear model: β=−1.54, 95% CI: −1.78 to −1.31, $P<2\times 10^{-16}$). For random samples of HA sequences, trees reconstructed from other segments simulated without reassortment had about 32.0% (interquartile range, IQR = 17.8% − 36.7%) as many SPRs as the real trees, on average. Specifically, the median SPR distance (number of rearrangements) was 98 (IQR 77.5 to 103) for real trees and 22 (16 to 27.5) for simulated trees (*n* = 35). These proportions were much lower for the control samples (3.8%, range 1.4% − 5.5%), supporting the hypothesis that phylogenetic uncertainty is the main driver of false positives. For these *n* = 7 samples, the median SPR distances were 143 (range 127–150) for real and 5 (2–7) for simulated trees. In addition, we obtained consistently higher SPR counts across segments for control samples (mean 140.7, range 127–150; Fig 3), which was likely driven by more complete coverage of the underlying evolutionary history of avian H5Nx isolates.

Even though we expect less phylogenetic discordance in the absence of reassortment, the number of false positives is unacceptably high (~30%) if the sequences have not been down-sampled to maximize evolutionary divergence. Therefore, it would be helpful to distinguish between real and spurious SPR events.

We investigated two characteristics of SPR events to determine whether SPRs arising from errors in phylogenetic reconstruction were distinguishable from those arising from reassortment. First, we examined the number of terminal nodes (tips) affected by the SPR, *i.e.*, subtree size. An unexpected result is that SPRs involving simulated trees tended to have the largest subtrees (Fig 4), despite significantly fewer subtrees being extracted from these comparisons.

**Fig 4:**
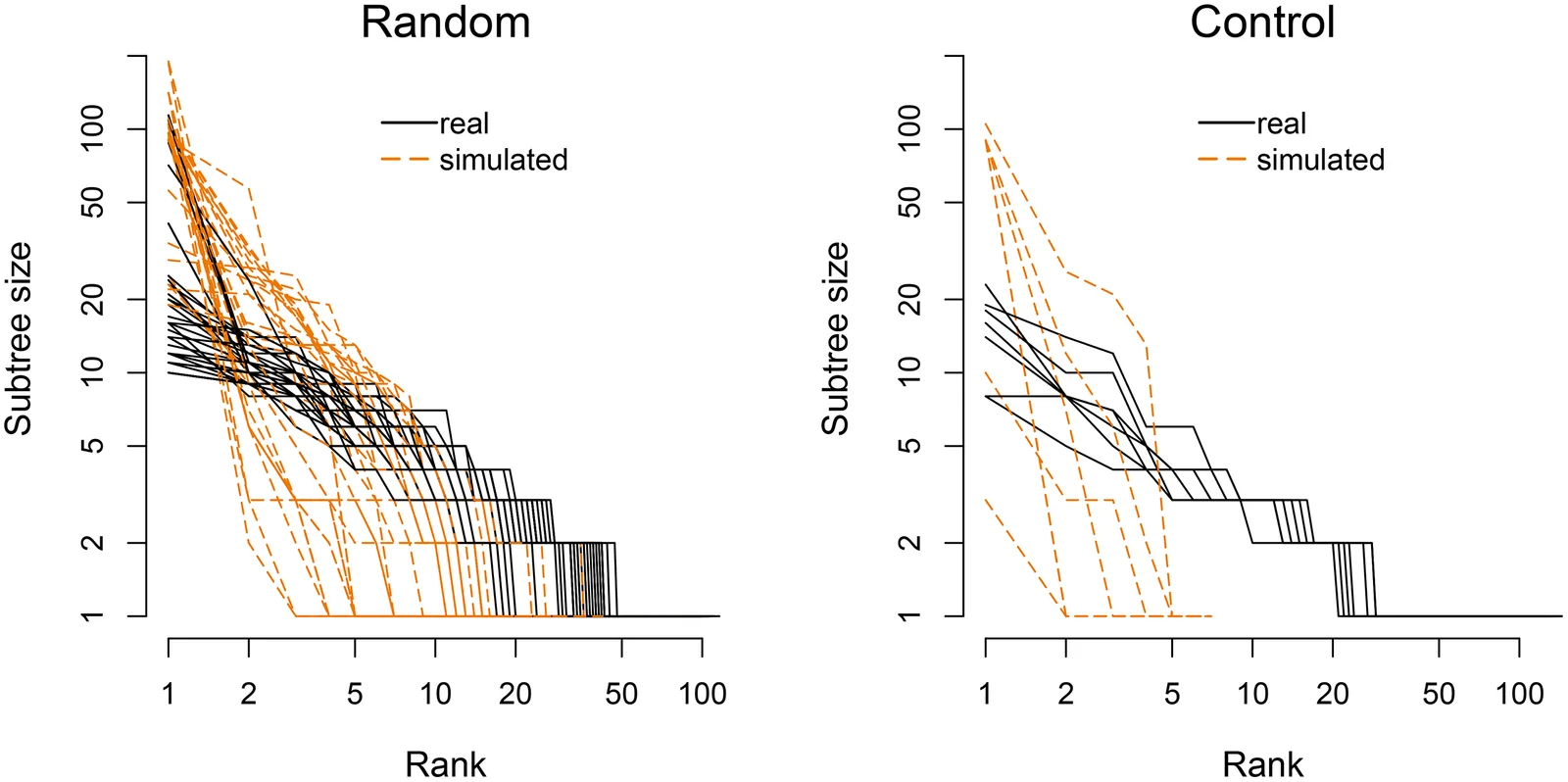
Log-log plots of subtree sizes for all SPR events reconstructed from random and control samples. Each line represents the sequence of subtrees for each segment and replicate (for random samples), sorted in decreasing order of size (number of tips). Solid (black) lines indicate subtrees in trees relating real segments, and dashed (orange) lines indicate simulations. The ranks of the ordered subtrees are plotted along the *x*-axis (*i.e.*, the largest subtree is rank 1), and subtree sizes are plotted along the *y*-axis.

This difference was statistically significant for random samples (paired Wilcoxon rank sum test, $P=4.16\times 10^{-6}$) and marginally significant for control samples (*P* = 0.051). On the other hand, there was no significant difference between real and simulated trees in the proportion of SPRs involving a single branch (binomial regression, *P* = 0.76).

Next, we quantified the distance between the prune and regraft points for all inferred SPR rearrangements. This task is not straightforward because these points exist on separate trees, so we approximated the path length by locating the smallest subtree containing the same tip labels as the descendants of the sibling node in a second tree, where the sibling node shares the same immediate ancestor as the subtree affected by the SPR. We refer to this path length as the sibling distance for a given SPR. Fig 5 summarizes the distributions of sibling distances for real and simulated trees in the random and control samples.

**Fig 5:**
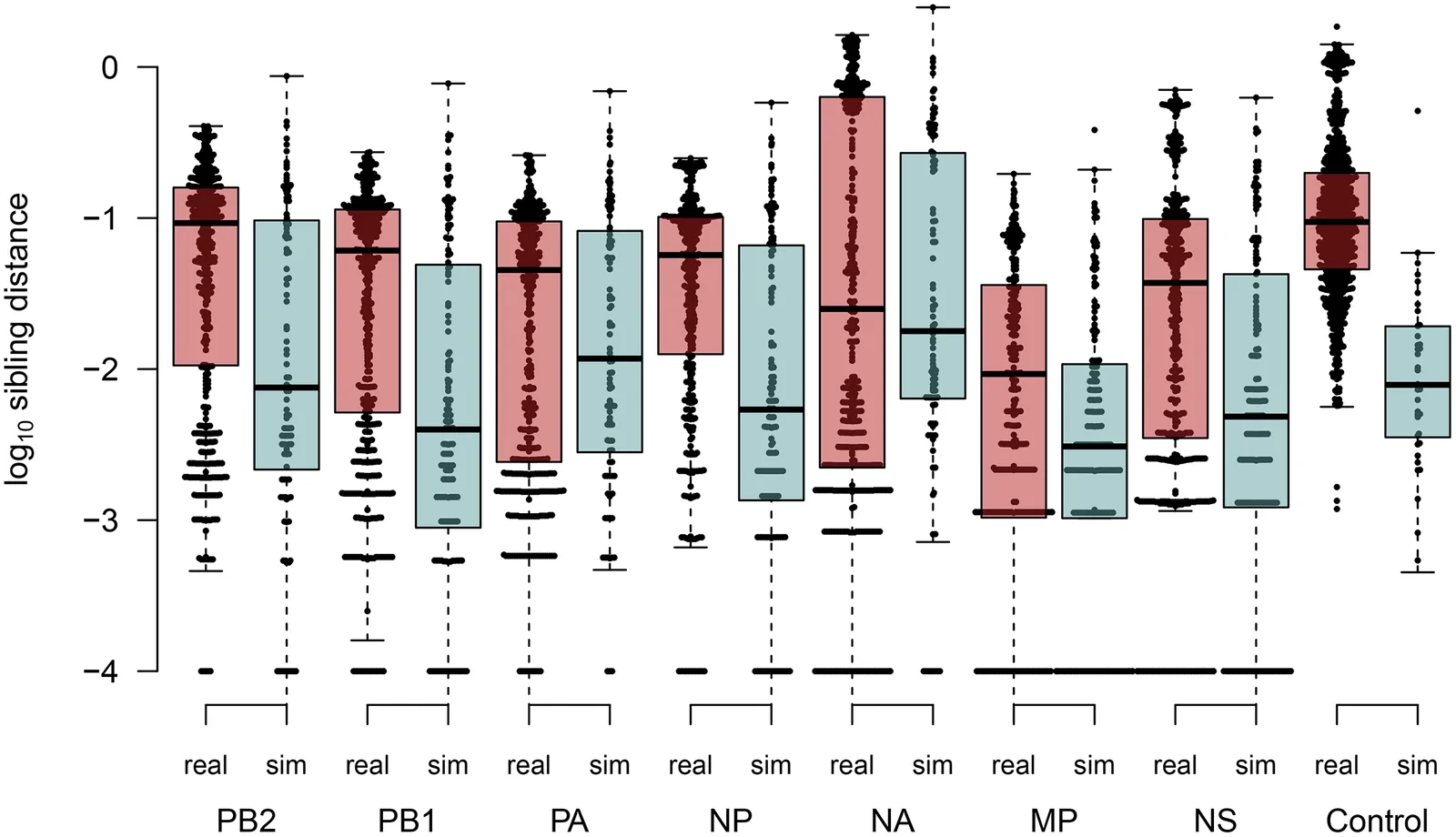
Boxplots showing the distribution of sibling distances (log_10_-transformed) between prune and regraft locations for SPR events across genome segments (PA, PB1, PB2, MP, NA, NP, and NS) for random samples, and pooled across segments for control samples. We added an arbitrarily small amount (10^–4^) to zero branch lengths to accommodate the log-transformation.

Overall, sibling distances were shorter in comparisons to simulated trees, with a median of 0.046 for real trees and 0.006 for simulated trees. If we assume that a reassortment event can occur between any two contemporaneous lineages in the population, then a tendency towards shorter sibling distances is more consistent with error in phylogenetic reconstruction. Sibling distances were more variable in comparisons between HA and NA trees (Fig 5), most likely because trees reconstructed from NA sequences were substantially larger than those from other segments; conversely, trees relating MP sequences were the shortest.

We used a gamma mixed-effects regression model with a log link to test the effect of data type (real or simulated) on sibling distance for random samples, including segment as a random effect. This model obtained a significant negative fixed effect of simulated data on sibling distance for random samples ($P=3.1\times 10^{-12}$, 95% CI = −0.53,−0.31). There was significant variation among segments (standard deviation, 95% CI = 0.28, 0.95). We did not find a significant association between subtree size and sibling distance (*P* = 0.13). Applying the same model to the control samples, we obtained a stronger effect of simulated data (*P* < 10^–12^, 95% CI = −2.58,−1.83) and a comparable amount of variation among segments (standard deviation 95% CI = 0.29, 1.02). The median sibling distance for control samples was 0.094 for real trees and 0.0078 for simulated trees. These results suggest that an effective way to reduce false positives in a reassortment analysis is to downsample the data to maximize sequence divergence. Additionally, SPRs with short sibling distances should be interpreted with caution, as the present analysis suggests they are often attributable to errors in phylogenetic reconstruction rather than to actual reassortment.

To complement our analysis of false-positive rates of inferring reassortment from SPR rearrangements, we also evaluated the false-negative rate. To accomplish this, we simulated reassortment events by directly applying SPR rearrangements to the phylogeny of the *n* = 213 most divergent HA sequences. First, we simulated 100 replicates with only one SPR rearrangement at a point selected uniformly at random in the tree. We used Espalier to identify SPRs between the original tree and each modified tree. Espalier recovered exactly the donor branch in every replicate (*i.e.*, no false negatives), corresponding to an event-level sensitivity of 100% (95% exact binomial confidence interval [CI]: 96.4–100%). Next, we evaluated a more challenging scenario by applying 10 independent SPR events across the 100 replicate trees, thereby simulating 1,000 reassortment events in total. Espalier recovered 915 donor branches (91.5% event-level sensitivity (95% CI: 89.6% – 93.2%), corresponding to a false-negative rate of 8.5% (95% CI: 6.9% – 10.4%). All simulated donor branches were correctly retrieved in 77 of 100 replicate trees (S4 Fig). These findings show that if the tree topology of the outbreak is known accurately, SPR-based methods can recover isolated reassortment events with high sensitivity. Evolutionary histories with more than one reassortment event, however, can partially obscure or overlap the topological signal from previous events, reducing the chance of recovering these events.

## Discussion

In this study, we evaluated two different methods to quantify the extent of reassortment from the topological discordance of two phylogenies: entanglement and the subtree prune regraft (SPR) distance. Entanglement is the total difference in the vertical placement of tip labels in a tanglegram that visualizes phylogenetic discordance. Our results are consistent with previous reports that entanglement can be misleading because the locations of tip labels are not directly proportional to topological congruence [23,37]. In other words, trees with very different topologies can have relatively low entanglement, and vice versa. We observed that phylogenetic discordance is more clearly resolved by SPR distances (Fig 3), which is arguably the most direct measure of reassortment that is readily available to us [20,38]. Moreover, we found that a substantial proportion of the discordance measured with either method can be attributed to phylogenetic reconstruction error instead of reassortment. For example, using the SPR distance to infer reassortment in simulated data yielded a false positive rate of about 30%. This finding is important because many studies of influenza virus genomes have relied on similar methods to identify extensive reassortment [22,39–41]. Our findings imply that the amount of reassortment may have been overestimated in some cases due to limitations in the accuracy of reconstructing phylogenies for different genomic segments [20].

Furthermore, we found that the impact of reconstruction error on the false positive rate for inferring reassortment is strongly influenced by how genome sequences have been sampled from the existing global variation. For example, random samples from the combined database of all published avian influenza H5Nx genomes produced significantly greater topological discordance than a sampling method that maximized the genetic diversity for a given number of genome sequences. Because closely related genomes contain fewer phylogenetically-informative substitutions, it becomes more difficult to resolve the internal branches of the phylogeny relating these genomes. Consequently, there is greater likelihood that independently reconstructed segment trees will be unreliable and differ from one another by chance, even when they share the same evolutionary history, as has been observed in other species [42–44]. We note that the rapid nucleotide substitution rates of AIV should provide a relative abundance of phylogenetically informative sites in genomic data within a timescale of years. These findings indicate that sampling design is a fundamental component of reassortment analyses. Initial subsampling based solely on divergence may exclude recent rearrangement events within the clade, since reassortment events in our false-negative analysis were simulated after subsampling. Maximizing sequence divergence is appropriate for inferring general patterns of reassortment and minimizes false-positive inferences due to phylogenetic reconstruction error. However, the benefit of using denser sampling, albeit at the expense of phylogenetic uncertainty, might be warranted in studies that focus on recent or lineage-specific reassortment. Moreover, identifying reassortment events for other segmented viruses with slower molecular clocks may be an even greater challenge [6].

Although the main objective of this study was to quantify the false-positive rate (specificity) of inferring reassortment from phylogenies, we also conducted simulations to evaluate the false-negative rate (sensitivity) of SPR-based methods under ideal conditions. SPR events were detected with 100% sensitivity when a single event per tree was introduced; however, when multiple rearrangements occurred within a single tree, sensitivity decreased. This decrease is expected, as successive SPR operations can be repeated or overlap, obscuring or attenuating the topological signal associated with preceding SPR operations. The SPR-based algorithms for detecting reassortment employ a data structure known as the maximum agreement forest (MAF) to calculate the minimum number of subtree rearrangements needed to transform one tree to another [36,45]. The MAF cannot be used to uniquely reconstruct the entire history of reassortment events in the case where multiple events have occurred [38]. Therefore, the false-negative rates observed here represent the worst-case scenario for the algorithm’s performance.

An interesting result from our simulations was that false-positive SPR events tended to be overrepresented in larger subtrees. Previous studies have demonstrated that deep ancestral relationships can be particularly difficult to resolve because of the limited amount of phylogenetic information available for resolving short internal branches, and the decay of phylogenetic signal in long terminal branches due to multiple substitutions and homoplasy [46]. Small errors in reconstructing deep ancestral relationships can propagate to the topology of entire descendant clades, particularly when the phylogenetic signal supporting basal nodes is weak [47]. Therefore, methods that detect reassortment events by inferring SPR rearrangements between trees are susceptible to assigning SPRs to deeper parts of the trees where there is limited precision in reconstructing internal branches, resulting in a skewed distribution of false positives. Accordingly, our finding that false positive reassortments tended to be associated with larger subtrees in simulations does not necessarily imply gross differences between the trees; this outcome can be produced by a small number of differences at short internal branches of the trees. Therefore, our simulation results suggest that large false-positive SPR events can occur even when the large topologies are somewhat similar, but differ at the short internal branches that are difficult to resolve.

### Previous work

Other studies in the literature have previously identified challenges in using phylogenetic discordance to infer reassortment events. Svinti *et al.* [20] identified several potential causes of phylogenetic discordance other than reassortment, including model misspecification, homoplasy, and incomplete sampling.

However, they did not attempt to measure the false positive rate caused by these processes, conceding that “we do not have a method for discovering the false rejection rate [for the null hypothesis of no reassortment] and indeed it is unlikely to be discoverable using current technology”. Neverov *et al.* [48] similarly noted that ‘phylogenetic noise’ may produce differences in phylogenies that result in spurious reassortment predictions. For instance, they observed that fewer reassortment events were identified in shorter or more conserved segments of influenza A virus H3N2 genomes. However, they did not quantitatively evaluate this effect. They additionally noted that reassortment events can be overlooked if none of the descendants of a parental lineage are sampled, or if multiple reassortments are nested within the same clade. These issues were further explored by Lin *et al.* [38], who described different mechanisms that can reduce the identifiability of reassortment events from comparing the trees of different genomic segments. As noted above, reassortments that involve an unsampled lineage cannot be reconstructed by comparing trees. In addition, comparing discordant trees can be affected by a ‘multiple hits’ problem in which a more recent reassortment event obscures other events that precede it. These issues have the opposite effect compared to phylogenetic uncertainty: they would cause the estimated number of SPR rearrangements to underestimate the actual extent of reassortment. On the other hand, Lin *et al.* did not examine the contribution of phylogenetic reconstruction error to the problem of identifying reassortments.

### Limitations

Our study focused on a specific program (Espalier [36]) for inferring reassortments through SPR rearrangements. Espalier uses a heuristic algorithm to compute the SPR distance more efficiently while retaining only strongly supported rearrangements. Thus, the algorithm employed by Espalier is approximate and can produce asymmetric distances, *i.e.*, d(A,B)≠d(B,A). A number of other programs have been developed to measure reassortment based on phylogenetic discordance, *e.g.*[20,27,49],. However, only a few of these programs remain available, particularly for inferring the minimum number of SPR rearrangements between trees. For example, SPRSupertrees [27] is a C++ program distributed under the GNU GPLv3 license that calculates the SPR distance, but its computing time is prohibitively long for phylogenetic trees with hundreds of branches or more. In addition, the website from which this program is distributed was not consistently accessible (last attempt October 15, 2025). Consequently, we selected Espalier as a currently available and actively maintained program that is representative of the SPR-based approach to detecting reassortment.

Espalier compares pairs of trees that are provided as user inputs, and are thereby processed without propagation of any uncertainty. This *post hoc* pairwise comparison of trees is also employed by several other programs for detecting reassortment, such as TreeKnit [50] and TreeSort [51]. TreeKnit is similar to Espalier in that it infers SPRs within the framework of the ancestral recombination / reassortment graph (ARG). An ARG is a graph-based representation of the evolutionary history of lineages relating different segments and individuals [52]. Lineages coalesce to common ancestors and split into alternate ancestral lineages due to reassortment. Another approach to detecting reassortment is to jointly infer the trees and network of reassortment events from input sequence alignments. This makes the uncertainty of reconstructing trees available for propagation to the end results. For example, CoalRe [53] uses Bayesian sampling to infer the joint posterior distribution of reassortment networks and trees. Instead of joint inference, Breassort [20] and GiRaF [54] employ a hierarchical approach, generating a posterior sample of trees from the sequence data before comparing trees for evidence of reassortment. However, Bayesian sampling methods have a greater computational cost due to the enormity of tree and graph spaces, and have been less frequently used to detect reassortment in larger influenza virus datasets.

Our study focused exclusively on the detection of reassortment from the topological discordance of phylogenies, which is the predominant approach for studying reassortment in influenza virus [55]. Reassortment can be detected on the basis of other criteria, however. For example, TreeSort [51] extracts the phylogenetic (tip-to-tip) distances between sequences in a tree, and compares them to the expected distribution under a null model without reassortment to identify outliers. It is possible for false positives to occur in this framework due to error in estimating branch lengths. On the other hand, tips may retain the same phylogenetic distance in trees with discordant topologies; ultrametric trees provide an extreme example of this scenario. Similarly, RIPPLES [56] uses parsimony to map substitutions to branches of a prespecified tree, and identifies branches with an excess number of substitutions as evidence of recombination in SARS-CoV-2 genomes. It resolves these outliers by splitting the sequence associated with the branch and assigning segments to different nodes in the tree by maximum parsimony. Because this method relies on a fixed input tree, it will be affected by errors in phylogenetic reconstruction. However, this approach has yet to be applied for detecting reassortment in influenza virus, and falls outside the scope of this study.

## Conclusion

Phylogenetic discordance is one of the most widely used methods to detect recombination in viruses with segmented genomes. In this work, we analyze genomic datasets from influenza A H5Nx viruses and compared them with simulated data representing the null hypothesis (absence of reassortment). We show that errors in reconstructing phylogenies alone can generate a considerable number of spurious rearrangements between trees that can be interpreted as evidence of reassortment of the corresponding segments. Thus, estimates of reassortment based on phylogenetic discordance should be interpreted with caution, especially when phylogenetic uncertainty is not taken into account. Our findings indicate that the sampling strategy plays a significant role in reducing the overestimation of false positives, since datasets that maximize genetic divergence exhibit significantly fewer spurious SPRs than datasets with random samples. Furthermore, our simulations indicate that spurious rearrangements tend to occupy similar locations in the respective trees. These results demonstrate that phylogenetic uncertainty should be incorporated into future studies for the detection and identification of reassortment events.

## Supporting information

S1 Fig Distributions of sample collection dates for hemagglutinin (HA) sequences from the NCBI GenBank and GISAID databases. (PDF)

S2 FigDiagram of the process for calculating the sibling distance of a subtree-prune-regraft (SPR) operation.For each subtree ($S_{j}$) in the maximum agreement forest, we identify the corresponding subtree in the segment 4 (HA) tree (*T*_4_) by locating the internal node that is ancestral to the smallest set of tip labels matching all labels in $S_{j}$. Next, we locate the sibling internal node in *T*_4_ ($s_{4}^{\left(j\right)}$) that shares an immediate common ancestor with $r_{4}^{\left(j\right)}$. We use the same label matching method to find the analogues of both $r_{4}^{\left(j\right)}$ and $s_{4}^{\left(j\right)}$ in a second tree relating either real ($T_{i}$) or simulated ($T_{i*}$) sequences of the *i*-th segment (where i≠4). Finally, we extract the total path length between these nodes ($r_{i}^{\left(j\right)}$ and $s_{i}^{\left(j\right)}$) as the sibling distance of the SPR.(PNG)

S3 Fig Tanglegram visualizing the correspondence between the phylogenetic trees of the HA and NA segments of influenza viruses, generated using the Python module *baltic* (https://github.com/evogytis/baltic). (A) Tanglegram based on real data, showing the relationship and potential reassortment events between matched strains for each segment. (B) Tanglegram comparison of the real HA tree with the simulated scaled NA tree. This panel shows potential reassortment events that arise in the simulated data.
(PNG)

S4 Fig Sensitivity of reassortment-event detection across simulations. Distribution of event-recovery sensitivity across 100 replicate trees, each containing 10 simulated subtree-prune-regraft (SPR) events. Sensitivity for each replicate was calculated as the proportion of simulated events for which the exact donor branch was recovered in the maximum agreement forest (MAF). Overall, 915 of 1,000 simulated events were recovered, corresponding to an event-level sensitivity of 91.5% and a false-negative rate of 8.5%. All 10 simulated events were recovered in 77 of the 100 replicate trees.
(PDF)
