# Supplementary material for: Assessing the impact of phylogenetic reconstruction error on genetic reassortment inference in segmented viruses
Source: PLoS One. 2026 Oct 22;21(10):e0360852. doi: 10.1371/journal.pone.0360852 (PMC13690219; doi:10.1371/journal.pone.0360852)
Supplement: Supplementary file 1 [file pone.0360852.s001.pdf]

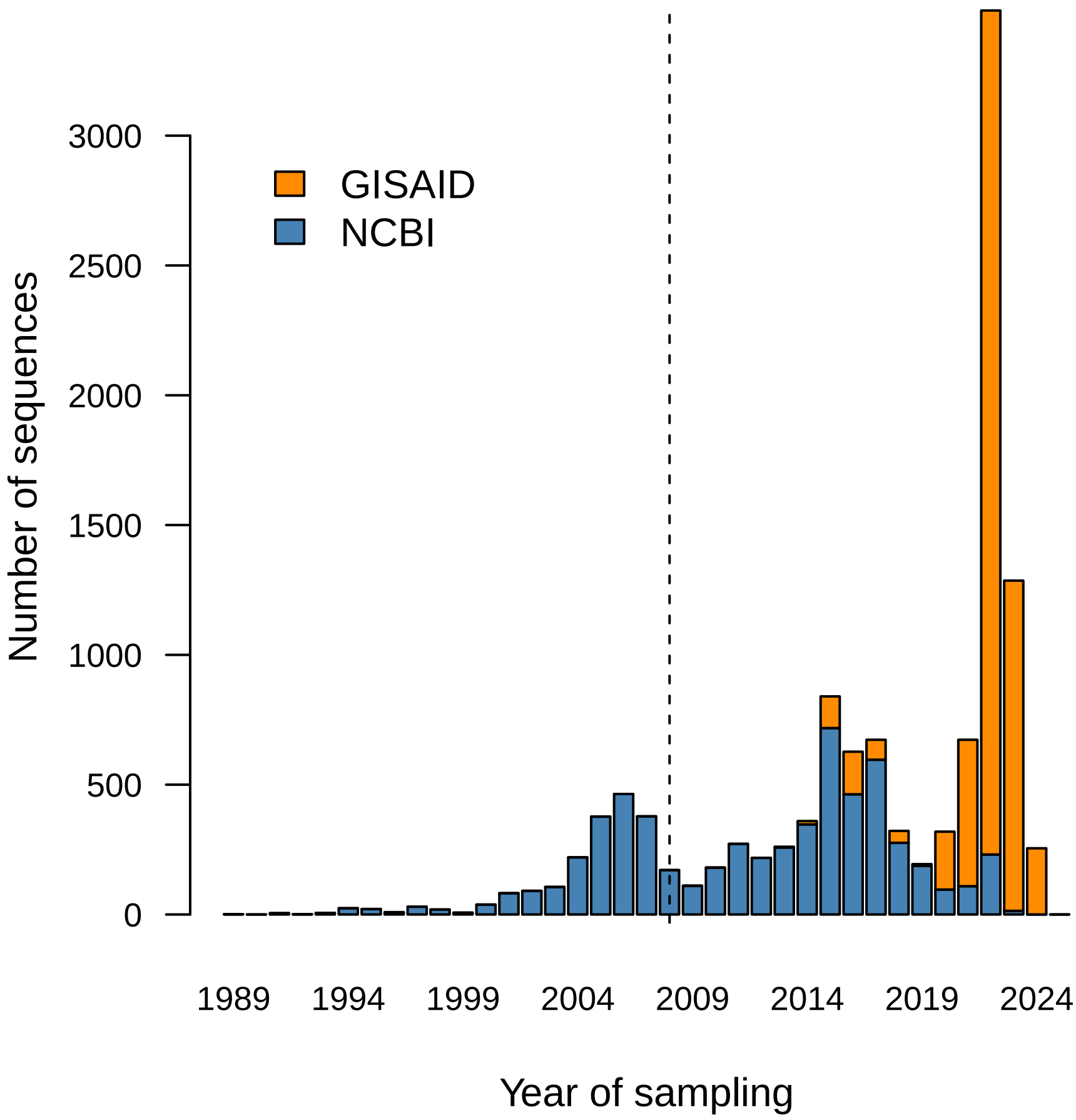

GISAID
NCBI
Number of sequences
0
500
1000
1500
2000
2500
3000
1989
1994
1999
2004
2009
2014
2019
2024
Year of sampling
