# Supplementary material for: Assessing the impact of phylogenetic reconstruction error on genetic reassortment inference in segmented viruses
Source: PLoS One. 2026 Oct 22;21(10):e0360852. doi: 10.1371/journal.pone.0360852 (PMC13690219; doi:10.1371/journal.pone.0360852)
Supplement: Supplementary file 2 [file pone.0360852.s004.pdf]

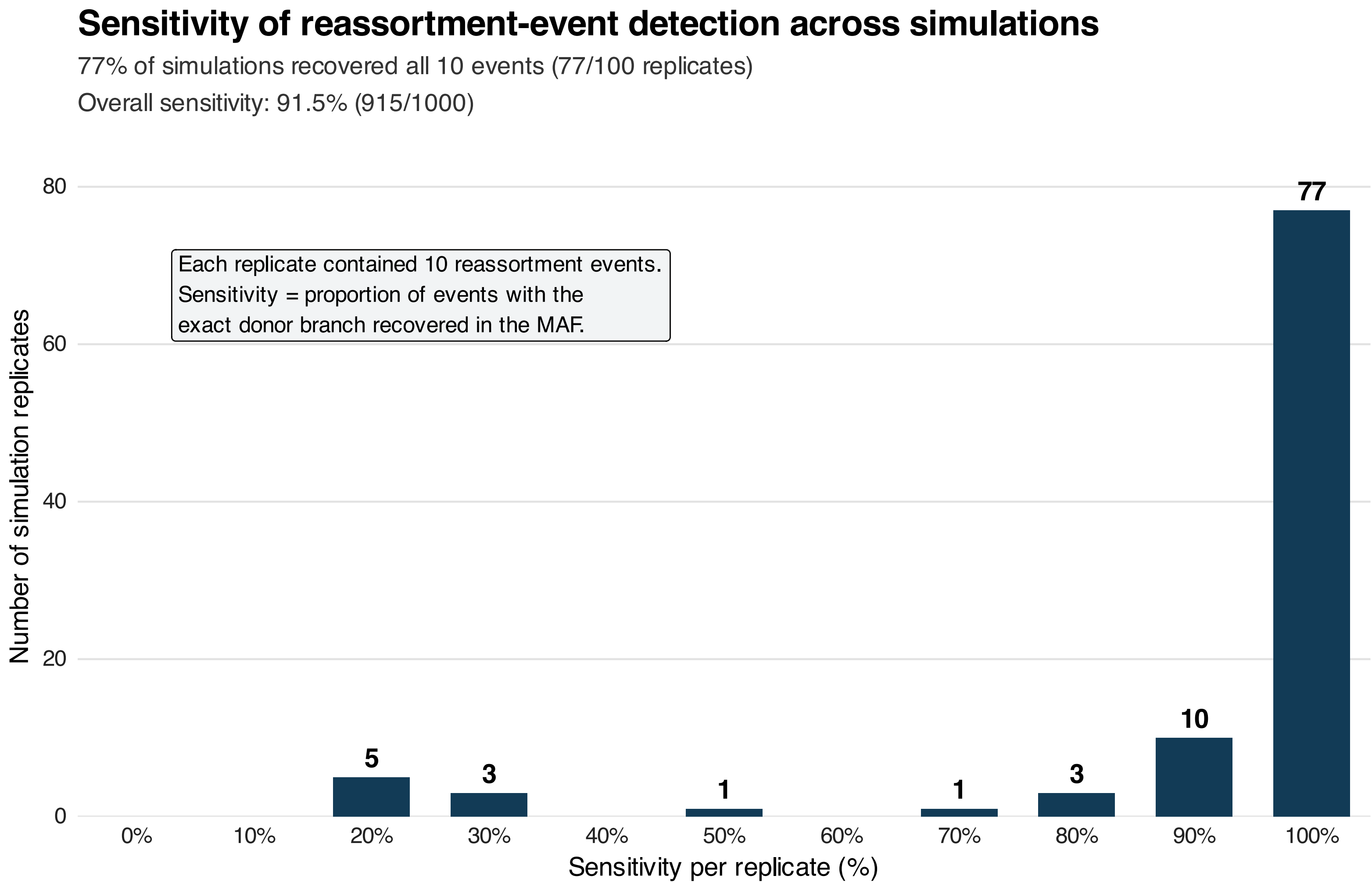
Sensitivity of reassortment-event detection across simulations
77% of simulations recovered all 10 events (77/100 replicates)
Overall sensitivity: 91.5% (915/1000)
Each replicate contained 10 reassortment events.
Sensitivity = proportion of events with the
exact donor branch recovered in the MAF.
Number of simulation replicates
0
20
40
60
80
5
3
1
1
3
10
77
0%
10%
20%
30%
40%
50%
60%
70%
80%
90%
100%
Sensitivity per replicate (%)
