# Supplementary material for: Assessing the impact of phylogenetic reconstruction error on genetic reassortment inference in segmented viruses
Source: PLoS One. 2026 Oct 22;21(10):e0360852. doi: 10.1371/journal.pone.0360852 (PMC13690219; doi:10.1371/journal.pone.0360852)
Supplement: Supplementary file 3 [file pone.0360852.s006.pdf]

Response to Reviewers

Manuscript ID: PONE-D-26-12074

Title: Assessing the impact of phylogenetic reconstruction error on genetic reassortment inference in segmented viruses

Hugo G. Castelán-Sánchez[1] and Art F. Y. Poon[1,2,3]

[1] Department of Pathology & Laboratory Medicine, Western University, London, Ontario, Canada
[2] Department of Microbiology & Immunology, Western University, London, Ontario, Canada
[3] Department of Computer Science, Western University, London, Ontario, Canada

Corresponding author:
Hugo G. Castelán-Sánchez (hcastela@uwo.ca)

Dear colleagues,

We thank the Academic Editor and Reviewer 1 for their careful evaluation of our manuscript and for their constructive comments. We have revised the manuscript extensively and addressed all concerns raised during the review process. Below we provide a detailed point-by-point response.

Academic Editor

Major comments

1. *"The manuscript comes with multiple typographical and alignment errors that significantly detract from its readability and professionalism. It must thoroughly undergo rigorous editorial review. Examples include 'muasured' instead of 'measured' in the abstract, etc. A meticulous line-by-line proofread of the entire text is required."*

   Response: We sincerely apologize for missing these errors in the previous version of the manuscript. We have performed a thorough line-by-line proof-reading of the manuscript to identify and rectify typographical, grammatical, formatting, and alignment issues. Specifically, we fixed the typographical errors (including the error in the abstract flagged by the editor), and improved sentence structure and consistency of terminology throughout.

2. *"The Discussion section is poorly crafted and improperly structured. Specifically, the first two paragraphs (page 15 onward, lines 2–26 and lines 1–17) read like a general background literature review rather than a critical synthesis of the current study's insights. Recommendation: The authors must completely restructure this section. The first paragraph of the Discussion should immediately articulate the main findings of this study. Subsequent paragraphs should then actively compare, contrast, and contextualize these results against existing literature, rather than rehashing broad scientific facts."*

   Response: We apologize for the deficiencies in the previous Discussion section. Based on the editor's points, we have extensively restructured the Discussion, so that it begins with an overview of the study's primary findings. This includes the finding that the uncertainty in phylogenetic reconstruction alone can produce substantial levels of spurious discordance between

trees, thereby inflating the estimated number of reassortment events from SPR distances. The subsequent paragraphs in the Discussion compare the results of the study with prior studies on influenza virus reassortment, addressing in particular the frequently-employed methods for detecting reassortment.

3. *"The study completely lacks a conclusion. The revised manuscript must feature a dedicated conclusion section that clearly encapsulates the primary takeaways of the study."*

   Response: A new Conclusion section has been added to summarize the key findings. This section emphasizes that errors in phylogenetic construction can lead to numerous false-positive reports of reassortment events. This section also describes the need to perform subsampling to reduce the number of SPR events, so that recombination events can be identified more accurately while avoiding false positives.

Specific Comments

1. *"The manuscript currently uses a page-by-page restarting line numbering format. For the benefit of the ongoing review and editorial process, the authors should configure the document settings to feature continuous line numbering from the title page through to the references."*

   Response: We have modified the formatting to use continuous line numbering throughout the document.

2. *"Abstract: The abstract lacks a defined objective. The revised manuscript should clearly state the objective of the study."*

   Response: In the revised abstract, we clearly define the objective of the study. We state explicitly that the purpose of the study is to evaluate how much the uncertainty in phylogenetic reconstruction can lead investigators to overestimate the number of reassortment events from phylogenetic discordance.

3. *"Result: on page 9 line 21, the authors state that entanglement values were 'significantly lower' based on a 95% confidence interval but fail to explicitly state the underlying statistical test performed, the exact test statistics, or the precise p-values. Furthermore, because phylogenetic edit distances (like SPR counts) are highly prone to overdispersion, the authors must clarify if their Poisson regression model was adjusted to ensure standard errors were not artificially deflated."*

   Response: We apologize for neglecting to provide this information. We have updated the manuscript to indicate the statistical tests performed, the resulting test statistics and the P-values along with the confidence intervals.

   Thank you for pointing out that phylogenetic edit distances are prone to overdispersion. We have since tested for overdispersion in the Poisson regression analysis of SPR counts. The Pearson dispersion parameter was 9.96, confirming substantial overdispersion ($\phi > 1$). We repeated the analysis using quasi-Poisson and negative binomial generalized linear models, which provide robust standard errors for overdispersed count data. Importantly, the principal result of the study remained unchanged: simulated trees exhibited significantly fewer SPR events than trees

reconstructed from real sequences (negative binomial model: $\beta = -1.54$, 95% CI: -1.78 to -1.31, $P < 2 \times 10^{-16}$). We have updated the manuscript to report these results and have replaced the original Poisson regression analysis with the more appropriate negative binomial model.

4. *"Result: On page 19 (lines 5–8), the authors present an 'unexpected result' showing that spurious, false-positive SPR moves in simulated data consistently involve the largest subtrees. This architectural anomaly is highly compelling but left entirely uninterpreted. The authors need to explicitly discuss (in the discussion section) the phylogenetic mechanism driving this (the subtree size paradox) – specifically, how minor resolution errors near deeply saturated, basal nodes of a tree can cause an algorithm to falsely infer massive macro-reassortment events."*

   Response: This is a very helpful comment from the Editor. We revised the Discussion by adding a new paragraph explaining that this apparent 'subtree size paradox' is most likely due to a phylogenetic mechanism. In particular, we consider that resolving deep phylogenetic relationships is challenging because of the lack of phylogenetically informative sites in short internal branches of the tree, as well as the loss of phylogenetic signal due to multiple substitutions and homoplasy on long terminal branches. Under these conditions, small reconstruction errors for deep internal nodes can cause the placement of entire descendant clades to change. Therefore, large SPR events should not always be assumed to represent large-scale reassortment events, because they may associated with internal nodes deep in the tree that are difficult to resolve.

5. *" Result: On page 13 (lines 7–10), the authors conclude that discard SPRs with short sibling distances. To make this manuscript truly impactful for the readers, this vague recommendation must be operationalized. The authors should propose a concrete mathematical threshold or operational rule of thumb (e.g., a specific substitution-per-site cutoff value) derived from their Gamma mixed-effects model or rephrase the sentence. "*

   Response: Thank you for your suggestion. We did not intend to propose a universal sibling-distance cutoff. Instead, we wanted to show that short sibling distances are more often linked to false-positive SPR inferences when reassortment is absent. The Gamma mixed-effects model shows a significant shift in sibling distance distributions between real and simulated datasets, but it was not designed to find the best classification threshold. Since sibling distance can depend on factors like sequence divergence, tree shape, and sampling design, we do not think a single substitution-per-site cutoff would be broadly useful or reliable. We have updated the text to make it clear that sibling distance should be seen as a relative measure of confidence, not a strict decision rule.

Reviewer 1

Major comments

1. *"In addition to looking at the false positive rate of SPR distance or tanglegram-based methods in identifying reassortment events, could the authors also simulate reassortment to assess the false negative rate of such methods?"*

Response: We appreciate this insightful question and agree that considering only false positives results in an incomplete assessment of subtree-prune-and-regraft (SPR) methods. To provide a more balanced evaluation, we also examined their ability to recover true reassortment events.

To do this, we introduced known reassortment events by direct SPR modification of the reference HA tree. By tracking the donor branch involved in each simulated SPR, we could directly compare the simulated reassortment history with the maximum agreement forest (MAF) inferred between the reference and reassortant trees.

We considered two simulation scenarios. In the first, we generated 100 replicate trees, each containing a single SPR event. In every case, *Espalier* recovered the correct donor branch, corresponding to a false negative rate of 0%. Next, we generated 100 replicate trees, each with 10 independent SPR events, for a total of 1,000 simulated reassortment events. We expected this second scenario to be more challenging because of potential overlaps between SPRs in the same tree. Under these conditions, *Espalier* correctly recovered 915 donor branches and missed 85, corresponding to an event-level false negative rate of 8.5%. Complete recovery of all simulated donor branches was achieved in 77 of the 100 replicate trees.

These findings show that when the underlying tree topology is known without error, SPR/MAF-based methods can recover isolated reassortment events with high accuracy. Their performance, however, declines as the number of reassortment events increases. A likely explanation is that successive SPR operations alter, overlap, or partially erase the topological signatures left by earlier events, making them progressively more difficult to detect. Furthermore, the MAF identifies the minimum set of topological cuts required to reconcile two trees rather than the complete sequence of historical SPR operations. As a result, when several reassortments have occurred, not every true event is expected to be recovered.

We have incorporated this new false-negative analysis into the revised manuscript. The Methods section now describes the simulation procedure, the criteria used to define exact donor-branch recovery, and the calculation of event-level sensitivity and false-negative rates. We also expanded the Discussion to emphasize that these estimates represent an upper bound on method performance because they were obtained using the true simulated trees. In empirical datasets, additional uncertainty introduced during sequence evolution and phylogenetic reconstruction is expected to further reduce the recovery of genuine reassortment events.

2. *"There appears to be evidence for some true within-clade reassortment that is lost when the data is downsampled to maximize divergence. Based on the Figure 5 plot, there are many more real sibling distance data points than simulated ones below 0.01. These short sibling distances are completely lost in the real control samples, presumably because of the lack of within-clade diversity. Could the authors try to quantify how much real signal is lost when downsampling the data to maximize sequence divergence? Perhaps by simulating reassortment in addition to simulating no reassortment, similar to what was mentioned above in comment #1, to obtain a measure of false negatives as a result of downsampling to maximize divergence. For people studying a less broad dataset, for example, to explore lineage-specific dynamics, would they still benefit from removing SPRs with short sibling distances?"*

   Response: We agree that there is a balance between minimizing phylogenetic reconstruction error and maximizing the resolution of recent within-clade reassortment events. To test this

question more extensively, another simulation analysis was carried out with the same maximally divergent reference tree (213 sequences) used in this study. Reassortment events, which were known, were placed on this tree to quantify the sensitivity of SPR reconstruction using the same sampling strategy we applied to our analyses.

The exact donor branch was recovered in all replicates (100% sensitivity) when a single SPR event was simulated per replicate. The method recovered 915 of 1000 simulated donor branches at the event level, with an event-level sensitivity of 91.5% and a false-negative rate of 8.5%, when 10 SPR events were simulated per replicate.

Results presented here show that SPR reconstruction remains highly sensitive even in more complex reassortment scenarios within the maximally divergent dataset examined. However, we agree that initial subsampling based solely on divergence may exclude recent rearrangement events within the clade, since these were simulated after subsampling.

We have now explained this restriction in the Discussion and make it explicit that maximizing sequence divergence is appropriate for inferring general patterns of reassortment and minimizes false positive inferences due to phylogenetic reconstruction error. However, the benefit of using denser sampling, albeit at the expense of phylogenetic uncertainty, might be warranted in studies that focus on recent or lineage-specific reassortment.

3. *"The authors point out that accounting for phylogenetic uncertainty is important for inferring reassortment patterns. Would the authors expect false positive reassortant calls to persist when using tools that model ancestral recombination graphs using parsimony with recombination distance cutoffs (e.g. https://www.nature.com/articles/s41586-022-05189-9 and https://academic.oup.com/mbe/article/42/8/msaf133/8227448), maximum likelihood (e.g. https://journals.plos.org/ploscompbiol/article?id=10.1371/journal.pcbi.1010394) or Bayesian (e.g. https://www.pnas.org/doi/abs/10.1073/pnas.1918304117) methods?"*

   Response: Thank you for raising this interesting question. The ancestral recombination / reassortment graph (ARG) represents a useful framework for inferring these events from data. Indeed, the program that was employed in our study (Espalier) uses ARGs to infer SPR rearrangements. However, using ARGs does not necessarily mean that the analysis accounts for phylogenetic uncertainty.

   First, we note that the first two studies cited by the reviewer do not really employ ARGs as a modeling framework. RIPPLES (Turakhia *et al.*, 2022) uses maximum parsimony to map substitutions to branches of a single fixed tree. TreeSort (Markin *et al.*, 2025) extracts the phylogenetic (tip-to-tip) distance between sequences from one tree to detect reassortment. In either case, there is no propagation of the uncertainty in phylogenetic reconstruction because fixed input trees are provided by the user. The criteria for identifying reassortment are also different. Our study evaluates the detection of reassortment from the discordance in the topologies of phylogenies. In contrast, TreeSort compares the phylogenetic distance to the expected null distribution under a strict molecular clock model. It is possible for false positives to occur due to error in estimating branch lengths, and thereby the phylogenetic distances. On the other hand, two trees may have different topologies and yet retain exactly the same phylogenetic distance between two sequences. Similarly, RIPPLES looks for branches in the tree that carry an unusually high number of substitutions, and resolves these outliers by assigning segments to different

nodes in the tree.

The maximum likelihood method (Treeknit; Barrat-Charlaix *et al.* 2022) requires the user to provide two trees as inputs for reconstructing the ARG. Consequently, it will not account for false positives due to phylogenetic reconstruction error. This issue is explicitly noted by the authors: "*Overall, the philosophy of TreeKnit is that the problem of poorly supported nodes should be dealt with before passing the trees to the algorithm.*"

Lastly, the Bayesian method cited by the reviewer (CoalRe; Müller *et al.*, 2020) jointly infers a posterior sample of trees and the network of reassortment events from the input sequence alignments. Hence the propagation of uncertainty in reconstructing phylogenies is explicitly incorporated into the analysis, and we should expect that this method will not suffer from false positives from reconstruction error. This is noted by the authors: "*As expected for methods that correctly take into account phylogenetic uncertainty, the posterior support decreases when lower evolutionary rates are used to simulate the sequences of the segments.*" However, this Bayesian approach also has a higher computational burden that is exacerbated by the enormous space of all possible ARGs, limiting its domain of application to smaller datasets.

We have added material to the Discussion section clarifying the similarities and limitations of these different approaches to inferring reassortment.

Minor Comments:

1. *"This wording in the methods is a bit confusing: 'progressively remove terminal branches in the HA tree whose length exceeded a cutoff' (page 6, line 4). This makes it sound like longer branches are also being removed, when in fact, from what I understand, only the shortest branches are removed."*

   Response: This sentence has been corrected to make clear we were progressively removing the shortest terminal branches until a target tree length (sum of all remaining branch lengths) was attained. This procedure was also clarified as being designed to keep the most genetically divergent sequences for the control set.

2. *"I do not understand the negative entanglement values seen and cited as Figure 2 (page 9, lines 21 and 24). The authors should describe more clearly the metric in parentheses, including the confidence intervals."*

   Response: We apologize for this misunderstanding. We were at fault for neglecting to indicate that the negative value reported on this line ("*Entanglement values were significantly lower for simulated trees (-0.12, ...*") was the difference in group means, *i.e.*, regression coefficient. In response to the editor's request, we now explicitly report the regression coefficient (β), the associated Wald t-statistic and P-value, and the 95% confidence interval.

3. *"Why does having eight segments imply that 99% of particles produced by a co-infected host cell will contain a reassortant genome (page 3, lines 2-4)?"*

   Response: We apologize for the lack of clarity. The influenza A virus (IAV) genome consists of eight segments. When a host cell has been multiply infected by two different IAV lineages, we

assume that the 'parentage' of the eight segments that are packaged into each new virus particle are independent random Bernoulli outcomes with probability 0.5 (*i.e.*, tosses of a fair coin). The only way that a virus particle contains a non-reassortant genome is if every segment comes from the same parental lineage. Thus, out of $2^8 = 256$ possible combinations of segments, 254 of them are reassortants and $254/256 = 0.9921875 \approx 0.99$. We have revised this text to provide a more accessible explanation and included a reference in support of this calculation.

Sincerely,

Hugo G. Castelán-Sánchez
on behalf of all authors
