# Supplementary material for: Assessing the impact of phylogenetic reconstruction error on genetic reassortment inference in segmented viruses
Source: PLoS One. 2026 Oct 22;21(10):e0360852. doi: 10.1371/journal.pone.0360852 (PMC13690219; doi:10.1371/journal.pone.0360852)
Supplement: Supplementary file 4 [file pone.0360852.s007.pdf]

Response to Reviewers

Manuscript ID: PONE-D-26-12074

Title: Assessing the impact of phylogenetic reconstruction error on genetic reassortment inference in segmented viruses

Hugo G. Castelán-Sánchez[1] and Art F. Y. Poon[1,2,3]

[1] Department of Pathology & Laboratory Medicine, Western University, London, Ontario, Canada
[2] Department of Microbiology & Immunology, Western University, London, Ontario, Canada
[3] Department of Computer Science, Western University, London, Ontario, Canada

Corresponding author:
Hugo G. Castelán-Sánchez (hcastela@uwo.ca)

Dear colleagues,

We have addressed the additional comments raised during this round of review. Below we provide a detailed point-by-point response.

Reviewer 1

Comments

1. *"Supplementary Material: Consider including a supplementary figure depicting the results of your false negative rate simulation experiment, alongside the existing description in the main text."*

   Response: We have added a new supplementary figure (Fig. S4) to visualize the results of the false-negative rate simulation experiment. The figure shows the distribution of event-recovery sensitivity across the 100 replicate trees containing 10 simulated SPR events. Overall, 915 of 1,000 simulated events were recovered, corresponding to an event-level sensitivity of 91.5% and a false-negative rate of 8.5%. All 10 simulated events were recovered in 77 of the 100 replicate trees. We have also cited Fig. S4 in the Results section.

2. *"The authors have expanded their discussion of subsampling to maximize sequence divergence. However, I recommend that the Discussion more explicitly reflect the trade-off between reducing phylogenetic reconstruction error and potentially excluding recent reassortment events occurring within closely related clades."*

   Response: We have revised the Discussion to make this trade-off more explicit. We have added the response we wrote in the reply to the reviewer in the discussion.

   The following text has been added to the Discussion:

   > *"Initial subsampling based solely on divergence may exclude recent rearrangement events within the clade, since reassortment events in our false-negative analysis were*

> *simulated after subsampling. Maximizing sequence divergence is appropriate for inferring general patterns of reassortment and minimizes false-positive inferences due to phylogenetic reconstruction error. However, the benefit of using denser sampling, albeit at the expense of phylogenetic uncertainty, might be warranted in studies that focus on recent or lineage-specific reassortment."*

Sincerely,

Hugo G. Castelán-Sánchez
on behalf of all authors
