# Supplementary material for: Assessing the impact of phylogenetic reconstruction error on genetic reassortment inference in segmented viruses
Source: PLoS One. 2026 Oct 22;21(10):e0360852. doi: 10.1371/journal.pone.0360852 (PMC13690219; doi:10.1371/journal.pone.0360852)
Supplement: Peer review history

# Peer review: Decision Letter 0

**DOI:** 10.1371/journal.pone.0360852.r001

**Authors:** Gadissa Hundie

15 Jun 2026

Dear Dr. Hugo G. Castelán-Sánchez,

Thank you for submitting your manuscript to PLOS ONE. After careful consideration, we feel that it has merit but does not fully meet PLOS ONE’s publication criteria as it currently stands. Therefore, we invite you to submit a revised version of the manuscript that addresses the points raised during the review process.

==============================

The manuscript addresses a highly relevant and frequently overlooked bottleneck in viral evolution, distinguishing genuine biological reassortment from artifacts caused by phylogenetic reconstruction error<source-footnote _nghost-ng-c2398278148="" ng-version="0.0.0-PLACEHOLDER"></source-footnote>. Your strategy of using simulated datasets under strict topological congruence to establish a false-positive baseline is robust and highly commended<source-footnote _nghost-ng-c2398278148="" ng-version="0.0.0-PLACEHOLDER"></source-footnote>. However, the manuscript lacks depth and quality in its current status and requires a **major revision**  before it can be considered for publication<source-footnote _nghost-ng-c2398278148="" ng-version="0.0.0-PLACEHOLDER"></source-footnote>. You are expected to address all points raised by both the reviewer and the academic editor in your revised manuscript point-by-point response<source-footnote _nghost-ng-c2398278148="" ng-version="0.0.0-PLACEHOLDER"></source-footnote><source-footnote _nghost-ng-c2398278148="" ng-version="0.0.0-PLACEHOLDER"></source-footnote><source-footnote _nghost-ng-c2398278148="" ng-version="0.0.0-PLACEHOLDER"></source-footnote><source-footnote _nghost-ng-c2398278148="" ng-version="0.0.0-PLACEHOLDER"></source-footnote>.

==============================

Please submit your revised manuscript by Jul 30 2026 11:59PM. If you will need more time than this to complete your revisions, please reply to this message or contact the journal office at plosone@plos.org. When you're ready to submit your revision, log on to https://www.editorialmanager.com/pone/ and select the 'Submissions Needing Revision' folder to locate your manuscript file.

- A letter that responds to each point raised by the academic editor and reviewer(s). You should upload this letter as a separate file labeled 'Response to Reviewers'.
- A marked-up copy of your manuscript that highlights changes made to the original version. You should upload this as a separate file labeled 'Revised Manuscript with Track Changes'.
- An unmarked version of your revised paper without tracked changes. You should upload this as a separate file labeled 'Manuscript'.

If you would like to make changes to your financial disclosure, please include your updated statement in your cover letter. Guidelines for resubmitting your figure files are available below the reviewer comments at the end of this letter.

If applicable, we recommend that you deposit your laboratory protocols in protocols.io to enhance the reproducibility of your results. Protocols.io assigns your protocol its own identifier (DOI) so that it can be cited independently in the future. For instructions see: https://journals.plos.org/plosone/s/submission-guidelines#loc-laboratory-protocols. Additionally, PLOS ONE offers an option for publishing peer-reviewed Lab Protocol articles, which describe protocols hosted on protocols.io. Read more information on sharing protocols at https://plos.org/protocols?utm_medium=editorial-email&utm_source=authorletters&utm_campaign=protocols.

As the corresponding author, your ORCID iD is verified in the submission system and will appear in the published article. PLOS supports the use of ORCID, and we encourage all coauthors to register for an ORCID iD and use it as well. Please encourage your coauthors to verify their ORCID iD within the submission system before final acceptance, as unverified ORCID iDs will not appear in the published article. *Only*  the individual author can complete the verification step; PLOS staff *cannot*  verify ORCID iDs on behalf of authors.

We look forward to receiving your revised manuscript.

Kind regards,

Gadissa Bedada Hundie, Ph.D

Academic Editor

PLOS One

Journal requirements:

When submitting your revision, we need you to address these additional requirements.

1. Please ensure that your manuscript meets PLOS ONE's style requirements, including those for file naming. The PLOS ONE style templates can be found at

https://journals.plos.org/plosone/s/file?id=wjVg/PLOSOne_formatting_sample_main_body.pdf and

https://journals.plos.org/plosone/s/file?id=ba62/PLOSOne_formatting_sample_title_authors_affiliations.pdf

2. Please note that PLOS One has specific guidelines on code sharing for submissions in which author-generated code underpins the findings in the manuscript. In these cases, we expect all author-generated code to be made available without restrictions upon publication of the work. Please review our guidelines at https://journals.plos.org/plosone/s/materials-and-software-sharing#loc-sharing-code and ensure that your code is shared in a way that follows best practice and facilitates reproducibility and reuse.

3. Please update your submission to use the PLOS LaTeX template. The template and more information on our requirements for LaTeX submissions can be found at http://journals.plos.org/plosone/s/latex.

4. We notice that your supplementary figures are included in the manuscript file. Please remove them and upload them with the file type 'Supporting Information'. Please ensure that each Supporting Information file has a legend listed in the manuscript after the references list.

5. We notice that your supplementary figures are uploaded with the file type 'Figure'. Please amend the file type to 'Supporting Information'. Please ensure that each Supporting Information file has a legend listed in the manuscript after the references list.

If the reviewer comments include a recommendation to cite specific previously published works, please review and evaluate these publications to determine whether they are relevant and should be cited. There is no requirement to cite these works unless the editor has indicated otherwise.

Additional Editor Comments:

The manuscript addresses an important, often overlooked problem in viral evolution: separating actual biological reassortment from artifacts of phylogenetic reconstruction error in segmented viruses. The overarching strategy using simulated datasets under strict topological congruence to establish a false-positive baseline is robust and highly relevant. However, the manuscript has widespread shortcomings that require extensive reorganization and development before it can be considered for publication.

Major Comments

1. The manuscript comes with multiple typographical and alignment errors that significantly detract from its readability and professionalism. It must thoroughly undergo rigorous editorial review. Examples include "muasured" instead of "measured" in the abstract, etc. A meticulous line-by-line proofread of the entire text is required.

2. The Discussion section is poorly crafted and improperly structured. Specifically, the first two paragraphs (page 15 onward, lines 2–26 and lines 1–17) read like a general background literature review rather than a critical synthesis of the current study's insights. Recommendation: The authors must completely restructure this section. The first paragraph of the Discussion should immediately articulate the main findings of this study. Subsequent paragraphs should then actively compare, contrast, and contextualize these results against existing literature, rather than rehashing broad scientific facts.

3. The study completely lacks a conclusion. The revised manuscript must feature a dedicated conclusion section that clearly encapsulates the primary takeaways of the study.

Specific Comments

• The manuscript currently uses a page-by-page restarting line numbering format. For the benefit of the ongoing review and editorial process, the authors should configure the document settings to feature continuous line numbering from the title page through to the references.

• Abstract: The abstract lacks a defined objective. The revised manuscript should clearly state the objective of the study.

• Result: on page 9 line 21, the authors state that entanglement values were "significantly lower" based on a 95% confidence interval but fail to explicitly state the underlying statistical test performed, the exact test statistics, or the precise p-values. Furthermore, because phylogenetic edit distances (like SPR counts) are highly prone to overdispersion, the authors must clarify if their Poisson regression model was adjusted to ensure standard errors were not artificially deflated.

• Result: On page 19 (lines 5–8), the authors present an "unexpected result" showing that spurious, false-positive SPR moves in simulated data consistently involve the largest subtrees. This architectural anomaly is highly compelling but left entirely uninterpreted. The authors need to explicitly discuss (in the discussion section) the phylogenetic mechanism driving this (the subtree size paradox)—specifically, how minor resolution errors near deeply saturated, basal nodes of a tree can cause an algorithm to falsely infer massive macro-reassortment events.

• Result: On page 13 (lines 7–10), the authors conclude that discard SPRs with short sibling distances. To make this manuscript truly impactful for the readers, this vague recommendation must be operationalized. The authors should propose a concrete mathematical threshold or operational rule of thumb (e.g., a specific substitution-per-site cutoff value) derived from their Gamma mixed-effects model or rephrase the sentence.

[Note: HTML markup is below. Please do not edit.]

Reviewers' comments:

Reviewer's Responses to Questions

**Comments to the Author**

1. Is the manuscript technically sound, and do the data support the conclusions?

Reviewer #1: Yes

**********

2. Has the statistical analysis been performed appropriately and rigorously?

Reviewer #1: Yes

**********

3. Have the authors made all data underlying the findings in their manuscript fully available?

The PLOS Data policy

Reviewer #1: Yes

**********

4. Is the manuscript presented in an intelligible fashion and written in standard English?

Reviewer #1: Yes

**********

Reviewer #1: In this manuscript, the authors test how well methods that quantify discordance between segment trees are able to reconstruct reassortment. By simulating the evolution of segments on top of a reference segment (HA), they find that the SPR distance as a measure of reassortment leads to an overestimation of reassortment events (large number of false positives). SPR distances, or more often tanglegrams are used to visualize the presence and extent of recombination. The manuscript is solid overall and shows that tanglegrams probably should not be relied upon to assess the presence of reassortment (and possibly by extension recombination). The focus of the paper is somewhat limited in that it largely focuses on false-positive reassortment calls and would ideally also involve a characterization of false negatives. There are few examples provided in which SPR distances have been used to quantify reassortment, so it is hard to assess whether this is a major issue. The authors do provide an approach for limiting the number of false positives by subsampling trees to maximize their genetic diversity, but could do more to discuss some of the possible downsides of this approach. Lastly, it would be helpful for the authors to address how these issues are handled in tools that model and account for uncertainty. Overall, the paper is technically sound and quantifies false positives well, but could benefit from additional characterization.

Major Comments:

1. In addition to looking at the false positive rate of SPR distance or tanglegram-based methods in identifying reassortment events, could the authors also simulate reassortment to assess the false negative rate of such methods?

2. There appears to be evidence for some true within-clade reassortment that is lost when the data is downsampled to maximize divergence. Based on the Figure 5 plot, there are many more real sibling distance data points than simulated ones below 0.01. These short sibling distances are completely lost in the real control samples, presumably because of the lack of within-clade diversity. Could the authors try to quantify how much real signal is lost when downsampling the data to maximize sequence divergence? Perhaps by simulating reassortment in addition to simulating no reassortment, similar to what was mentioned above in comment #1, to obtain a measure of false negatives as a result of downsampling to maximize divergence. For people studying a less broad dataset, for example to explore lineage-specific dynamics, would they still benefit from removing SPRs with short sibling distances?

3. The authors point out that accounting for phylogenetic uncertainty is important for inferring reassortment patterns. Would the authors expect false positive reassortant calls to persist when using tools that model ancestral recombination graphs using parsimony with recombination distance cutoffs (e.g. https://www.nature.com/articles/s41586-022-05189-9 and https://academic.oup.com/mbe/article/42/8/msaf133/8227448), maximum likelihood (e.g. https://journals.plos.org/ploscompbiol/article?id=10.1371/journal.pcbi.1010394) or Bayesian (e.g. https://www.pnas.org/doi/abs/10.1073/pnas.1918304117) methods?

Minor Comments:

1. It would be useful for the authors to explicitly discuss examples in the literature where SPR distance or tanglegrams have been used to identify reassortment events. How frequently are these methods utilized currently for determining whether reassortment has occurred? The manuscripts cited in the introduction establishing that "the comparative analysis of IAV genome sequences plays an important role in measuring the frequency of reassortment and detecting the emergence of new reassortant viruses" (page 3, lines 9-11) are quite broad, and it would be beneficial to instead specifically cite examples here where SPR distance or tanglegrams were used to identify reassortants.

2. This wording in the methods is a bit confusing: "progressively remove terminal branches in the HA tree whose length exceeded a cutoff" (page 6, line 4). This makes it sound like longer branches are also being removed, when in fact from what I understand only the shortest branches are removed.

3. I do not understand the negative entanglement values seen and cited as Figure 2 (page 9, lines 21 and 24). The authors should describe more clearly the metric in parentheses, including the confidence intervals.

4. Why does having eight segments imply that 99% of particles produced by a co-infected host cell will contain a reassortant genome (page 3, lines 2-4)?

**********

what does this mean?). If published, this will include your full peer review and any attached files.

If you choose “no”, your identity will remain anonymous but your review may still be made public.

**Do you want your identity to be public for this peer review?**  For information about this choice, including consent withdrawal, please see our Privacy Policy

Reviewer #1: No

**********

[NOTE: If reviewer comments were submitted as an attachment file, they will be attached to this email and accessible via the submission site. Please log into your account, locate the manuscript record, and check for the action link "View Attachments". If this link does not appear, there are no attachment files.]

To ensure your figures meet our technical requirements, please review our figure guidelines: https://journals.plos.org/plosone/s/figures

You may also use PLOS’s free figure tool, NAAS, to help you prepare publication quality figures: https://journals.plos.org/plosone/s/figures#loc-tools-for-figure-preparation.

NAAS will assess whether your figures meet our technical requirements by comparing each figure against our figure specifications.

# Author comment: Author response to Decision Letter 1

**DOI:** 10.1371/journal.pone.0360852.r002

19 Aug 2026

Manuscript ID: PONE-D-26-12074

Title: Assessing the impact of phylogenetic reconstruction error on genetic reassortment inference in segmented viruses

Hugo G. Castelán-Sánchez¹ and Art F. Y. Poon¹,²,³

¹ Department of Pathology & Laboratory Medicine, Western University, London, Ontario, Canada

² Department of Microbiology & Immunology, Western University, London, Ontario, Canada

³ Department of Computer Science, Western University, London, Ontario, Canada

Corresponding author: Hugo G. Castelán-Sánchez (hcastela@uwo.ca)

Dear colleagues,

We thank the Academic Editor and Reviewer 1 for their careful evaluation of our manuscript and for their constructive comments. We have revised the manuscript extensively and addressed all concerns raised during the review process. Below we provide a detailed point-by-point response.

Academic Editor

Major comments

1. “The manuscript comes with multiple typographical and alignment errors that significantly detract from its readability and professionalism. It must thoroughly undergo rigorous editorial review. Examples include ‘muasured’ instead of ‘measured’ in the abstract, etc. A meticulous line-by-line proofread of the entire text is required.”

Response: We sincerely apologize for missing these errors in the previous version of the manuscript. We have performed a thorough line-by-line proof-reading of the manuscript to identify and rectify typographical, grammatical, formatting, and alignment issues. Specifically, we fixed the typographical errors (including the error in the abstract flagged by the editor), and improved sentence structure and consistency of terminology throughout.

2. “The Discussion section is poorly crafted and improperly structured. Specifically, the first two paragraphs (page 15 onward, lines 2–26 and lines 1–17) read like a general background literature review rather than a critical synthesis of the current study’s insights. Recommendation: The authors must completely restructure this section. The first paragraph of the Discussion should immediately articulate the main findings of this study. Subsequent paragraphs should then actively compare, contrast, and contextualize these results against existing literature, rather than rehashing broad scientific facts.”

Response: We apologize for the deficiencies in the previous Discussion section. Based on the editor’s points, we have extensively restructured the Discussion, so that it begins with an overview of the study’s primary findings. This includes the finding that the uncertainty in phylogenetic reconstruction alone can produce substantial levels of spurious discordance between trees, thereby inflating the estimated number of reassortment events from SPR distances. The subsequent paragraphs in the Discussion compare the results of the study with prior studies on influenza virus reassortment, addressing in particular the frequently-employed methods for detecting reassortment.

3. “The study completely lacks a conclusion. The revised manuscript must feature a dedicated conclusion section that clearly encapsulates the primary takeaways of the study.”

Response: A new Conclusion section has been added to summarize the key findings. This section emphasizes that errors in phylogenetic construction can lead to numerous false-positive reports of reassortment events. This section also describes the need to perform subsampling to reduce the number of SPR events, so that recombination events can be identified more accurately while avoiding false positives.

Specific Comments

1. “The manuscript currently uses a page-by-page restarting line numbering format. For the benefit of the ongoing review and editorial process, the authors should configure the document settings to feature continuous line numbering from the title page through to the references.”

Response: We have modified the formatting to use continuous line numbering throughout the document.

2. “Abstract: The abstract lacks a defined objective. The revised manuscript should clearly state the objective of the study.”

Response: In the revised abstract, we clearly define the objective of the study. We state explicitly that the purpose of the study is to evaluate how much the uncertainty in phylogenetic reconstruction can lead investigators to overestimate the number of reassortment events from phylogenetic discordance.

3. “Result: on page 9 line 21, the authors state that entanglement values were ‘significantly lower’ based on a 95% confidence interval but fail to explicitly state the underlying statistical test performed, the exact test statistics, or the precise p-values. Furthermore, because phylogenetic edit distances (like SPR counts) are highly prone to overdispersion, the authors must clarify if their Poisson regression model was adjusted to ensure standard errors were not artificially deflated.”

Response: We apologize for neglecting to provide this information. We have updated the manuscript to indicate the statistical tests performed, the resulting test statistics and the P-values along with the confidence intervals.

Thank you for pointing out that phylogenetic edit distances are prone to overdispersion. We have since tested for overdispersion in the Poisson regression analysis of SPR counts. The Pearson dispersion parameter was 9.96, confirming substantial overdispersion (φ > 1). We repeated the analysis using quasi-Poisson and negative binomial generalized linear models, which provide robust standard errors for overdispersed count data. Importantly, the principal result of the study remained unchanged: simulated trees exhibited significantly fewer SPR events than trees reconstructed from real sequences (negative binomial model: β = −1.54, 95% CI: −1.78 to −1.31, P < 2×10−16). We have updated the manuscript to report these results and have replaced the original Poisson regression analysis with the more appropriate negative binomial model.

4. “Result: On page 19 (lines 5–8), the authors present an ‘unexpected result’ showing that spurious, false-positive SPR moves in simulated data consistently involve the largest subtrees. This architectural anomaly is highly compelling but left entirely uninterpreted. The authors need to explicitly discuss (in the discussion section) the phylogenetic mechanism driving this (the subtree size paradox) – specifically, how minor resolution errors near deeply saturated, basal nodes of a tree can cause an algorithm to falsely infer massive macro-reassortment events.”

Response: This is a very helpful comment from the Editor. We revised the Discussion by adding a new paragraph explaining that this apparent ‘subtree size paradox’ is most likely due to a phylogenetic mechanism. In particular, we consider that resolving deep phylogenetic relationships is challenging because of the lack of phylogenetically informative sites in short internal branches of the tree, as well as the loss of phylogenetic signal due to multiple substitutions and homoplasy on long terminal branches. Under these conditions, small reconstruction errors for deep internal nodes can cause the placement of entire descendant clades to change. Therefore, large SPR events should not always be assumed to represent large-scale reassortment events, because they may associated with internal nodes deep in the tree that are difficult to resolve.

5. “Result: On page 13 (lines 7–10), the authors conclude that discard SPRs with short sibling distances. To make this manuscript truly impactful for the readers, this vague recommendation must be operationalized. The authors should propose a concrete mathematical threshold or operational rule of thumb (e.g., a specific substitution-per-site cutoff value) derived from their Gamma mixed-effects model or rephrase the sentence.”

Response: Thank you for your suggestion. We did not intend to propose a universal sibling-distance cutoff. Instead, we wanted to show that short sibling distances are more often linked to false-positive SPR inferences when reassortment is absent. The Gamma mixed-effects model shows a significant shift in sibling distance distributions between real and simulated datasets, but it was not designed to find the best classification threshold. Since sibling distance can depend on factors like sequence divergence, tree shape, and sampling design, we do not think a single substitution-per-site cutoff would be broadly useful or reliable. We have updated the text to make it clear that sibling distance should be seen as a relative measure of confidence, not a strict decision rule.

Reviewer 1

Major comments

1. “In addition to looking at the false positive rate of SPR distance or tanglegram-based methods in identifying reassortment events, could the authors also simulate reassortment to assess the false negative rate of such methods?”

Response: We appreciate this insightful question and agree that considering only false positives results in an incomplete assessment of subtree-prune-and-regraft (SPR) methods. To provide a more balanced evaluation, we also examined their ability to recover true reassortment events. To do this, we introduced known reassortment events by direct SPR modification of the reference HA tree. By tracking the donor branch involved in each simulated SPR, we could directly compare the simulated reassortment history with the maximum agreement forest (MAF) inferred between the reference and reassortant trees.

We considered two simulation scenarios. In the first, we generated 100 replicate trees, each containing a single SPR event. In every case, Espalier recovered the correct donor branch, corresponding to a false negative rate of 0%. Next, we generated 100 replicate trees, each with 10 independent SPR events, for a total of 1,000 simulated reassortment events. We expected this second scenario to be more challenging because of potential overlaps between SPRs in the same tree. Under these conditions, Espalier correctly recovered 915 donor branches and missed 85, corresponding to an event-level false negative rate of 8.5%. Complete recovery of all simulated donor branches was achieved in 77 of the 100 replicate trees.

These findings show that when the underlying tree topology is known without error, SPR/MAF-based methods can recover isolated reassortment events with high accuracy. Their performance, however, declines as the number of reassortment events increases. A likely explanation is that successive SPR operations alter, overlap, or partially erase the topological signatures left by earlier events, making them progressively more difficult to detect. Furthermore, the MAF identifies the minimum set of topological cuts required to reconcile two trees rather than the complete sequence of historical SPR operations. As a result, when several reassortments have occurred, not every true event is expected to be recovered.

We have incorporated this new false-negative analysis into the revised manuscript. The Methods section now describes the simulation procedure, the criteria used to define exact donor-branch recovery, and the calculation of event-level sensitivity and false-negative rates. We also expanded the Discussion to emphasize that these estimates represent an upper bound on method performance because they were obtained using the true simulated trees. In empirical datasets, additional uncertainty introduced during sequence evolution and phylogenetic reconstruction is expected to further reduce the recovery of genuine reassortment events.

2. “There appears to be evidence for some true within-clade reassortment that is lost when the data is downsampled to maximize divergence. Based on the Figure 5 plot, there are many more real sibling distance data points than simulated ones below 0.01. These short sibling distances are completely lost in the real control samples, presumably because of the lack of within-clade diversity. Could the authors try to quantify how much real signal is lost when downsampling the data to maximize sequence divergence? Perhaps by simulating reassortment in addition to simulating no reassortment, similar to what was mentioned above in comment #1, to obtain a measure of false negatives as a result of downsampling to maximize divergence. For people studying a less broad dataset, for example, to explore lineage-specific dynamics, would they still benefit from removing SPRs with short sibling distances?”

Response: We agree that there is a balance between minimizing phylogenetic reconstruction error and maximizing the resolution of recent within-clade reassortment events. To test this question more extensively, another simulation analysis was carried out with the same maximally divergent reference tree (213 sequences) used in this study. Reassortment events, which were known, were placed on this tree to quantify the sensitivity of SPR reconstruction using the same sampling strategy we applied to our analyses.

The exact donor branch was recovered in all replicates (100% sensitivity) when a single SPR event was simulated per replicate. The method recovered 915 of 1000 simulated donor branches at the event level, with an event-level sensitivity of 91.5% and a false-negative rate of 8.5%, when 10 SPR events were simulated per replicate.

Results presented here show that SPR reconstruction remains highly sensitive even in more complex reassortment scenarios within the maximally divergent dataset examined. However, we agree that initial subsampling based solely on divergence may exclude recent rearrangement events within the clade, since these were simulated after subsampling.

We have now explained this restriction in the Discussion and make it explicit that maximizing sequence divergence is appropriate for inferring general patterns of reassortment and minimizes false positive inferences due to phylogenetic reconstruction error. However, the benefit of using denser sampling, albeit at the expense of phylogenetic uncertainty, might be warranted in studies that focus on recent or lineage-specific reassortment.

3. “The authors point out that accounting for phylogenetic uncertainty is important for inferring reassortment patterns. Would the authors expect false positive reassortant calls to persist when using tools that model ancestral recombination graphs using parsimony with recombination distance cutoffs, maximum likelihood, or Bayesian methods?”

Response: Thank you for raising this interesting question. The ancestral recombination / reassortment graph (ARG) represents a useful framework for inferring these events from data. Indeed, the program that was employed in our study (Espalier) uses ARGs to infer SPR rearrangements. However, using ARGs does not necessarily mean that the analysis accounts for phylogenetic uncertainty.

First, we note that the first two studies cited by the reviewer do not really employ ARGs as a modeling framework. RIPPLES (Turakhia et al., 2022) uses maximum parsimony to map substitutions to branches of a single fixed tree. TreeSort (Markin et al., 2025) extracts the phylogenetic (tip-to-tip) distance between sequences from one tree to detect reassortment. In either case, there is no propagation of the uncertainty in phylogenetic reconstruction because fixed input trees are provided by the user. The criteria for identifying reassortment are also different. Our study evaluates the detection of reassortment from the discordance in the topologies of phylogenies. In contrast, TreeSort compares the phylogenetic distance to the expected null distribution under a strict molecular clock model. It is possible for false positives to occur due to error in estimating branch lengths, and thereby the phylogenetic distances. On the other hand, two trees may have different topologies and yet retain exactly the same phylogenetic distance between two sequences. Similarly, RIPPLES looks for branches in the tree that carry an unusually high number of substitutions, and resolves these outliers by assigning segments to different nodes in the tree.

The maximum likelihood method (Treeknit; Barrat-Charlaix et al. 2022) requires the user to provide two trees as inputs for reconstructing the ARG. Consequently, it will not account for false positives due to phylogenetic reconstruction error. This issue is explicitly noted by the authors: “Overall, the philosophy of TreeKnit is that the problem of poorly supported nodes should be dealt with before passing the trees to the algorithm.”

Lastly, the Bayesian method cited by the reviewer (CoalRe; Müller et al., 2020) jointly infers a posterior sample of trees and the network of reassortment events from the input sequence alignments. Hence the propagation of uncertainty in reconstructing phylogenies is explicitly incorporated into the analysis, and we should expect that this method will not suffer from false positives from reconstruction error. This is noted by the authors: “As expected for methods that correctly take into account phylogenetic uncertainty, the posterior support decreases when lower evolutionary rates are used to simulate the sequences of the segments.” However, this Bayesian approach also has a higher computational burden that is exacerbated by the enormous space of all possible ARGs, limiting its domain of application to smaller datasets.

We have added material to the Discussion section clarifying the similarities and limitations of these different approaches to inferring reassortment.

Minor Comments:

1. “This wording in the methods is a bit confusing: ‘progressively remove terminal branches in the HA tree whose length exceeded a cutoff’ (page 6, line 4). This makes it sound like longer branches are also being removed, when in fact, from what I understand, only the shortest branches are removed.”

Response: This sentence has been corrected to make clear we were progressively removing the shortest terminal branches until a target tree length (sum of all remaining branch lengths) was attained. This procedure was also clarified as being designed to keep the most genetically divergent sequences for the control set.

2. “I do not understand the negative entanglement values seen and cited as Figure 2 (page 9, lines 21 and 24). The authors should describe more clearly the metric in parentheses, including the confidence intervals.”

Response: We apologize for this misunderstanding. We were at fault for neglecting to indicate that the negative value reported on this line (“Entanglement values were significantly lower for simulated trees (−0.12, …”) was the difference in group means, i.e., regression coefficient. In response to the editor’s request, we now explicitly report the regression coefficient (β), the associated Wald t-statistic and P-value, and the 95% confidence interval.

3. “Why does having eight segments imply that 99% of particles produced by a co-infected host cell will contain a reassortant genome (page 3, lines 2-4)?”

Response: We apologize for the lack of clarity. The influenza A virus (IAV) genome consists of eight segments. When a host cell has been multiply infected by two different IAV lineages, we assume that the ‘parentage’ of the eight segments that are packaged into each new virus particle are independent random Bernoulli outcomes with probability 0.5 (i.e., tosses of a fair coin). The only way that a virus particle contains a non-reassortant genome is if every segment comes from the same parental lineage. Thus, out of 2⁸ = 256 possible combinations of segments, 254 of them are reassortants and 254/256 = 0.9921875 ≈ 0.99. We have revised this text to provide a more accessible explanation and included a reference in support of this calculation.

Sincerely,

Hugo G. Castelán-Sánchez

on behalf of all authors

Attachment Submitted filename: letter_reponses_tex.pdf

# Peer review: Decision Letter 1

**DOI:** 10.1371/journal.pone.0360852.r003

**Authors:** Gadissa Hundie

17 Sep 2026

PLOS One

Dear Dr. Hugo G. Castelán-Sánchez,

Thank you for submitting your manuscript to PLOS One. After careful consideration, we feel that it has merit but does not fully meet PLOS One’s publication criteria as it currently stands. Therefore, we invite you to submit a revised version of the manuscript that addresses the points raised during the review process.

==============================

Dear Dr. Hugo G. Castelán-Sánchez

Thank you for submitting your revised manuscript "PONE-D-26-12074 R1" to *PLOS ONE* .

I have evaluated the revised manuscript alongside the reviewer's comments. I am pleased to inform you that your manuscript is conceptually ready for publication, subject to a few minor text and formatting adjustments.

The reviewer has recommended acceptance and noted significant improvements in readability and methodology. Before final acceptance, please address the following minor points raised by the reviewer:

1. Supplementary Material: Consider including a supplementary figure depicting the results of your false negative rate simulation experiment, alongside the existing description in the main text.
2. Discussion Clarification: Expand slightly on the limitations and trade-offs associated with your sequence subsampling strategy (specifically, maximizing sequence divergence versus capturing recent reassortment events within clades). Ensure that lines 514–522 explicitly reflect these trade-offs as suggested by the reviewer.

Sincerely,

Dr. Gadissa Bedada Hundie

Academic Editor, *PLOS ONE*

==============================

Please submit your revised manuscript by Nov 01 2026 11:59PM. If you will need more time than this to complete your revisions, please reply to this message or contact the journal office at plosone@plos.org. When you're ready to submit your revision, log on to https://www.editorialmanager.com/pone/ and select the 'Submissions Needing Revision' folder to locate your manuscript file.

- A letter that responds to each point raised by the academic editor and reviewer(s). You should upload this letter as a separate file labeled 'Response to Reviewers'.
- A marked-up copy of your manuscript that highlights changes made to the original version. You should upload this as a separate file labeled 'Revised Manuscript with Track Changes'.
- An unmarked version of your revised paper without tracked changes. You should upload this as a separate file labeled 'Manuscript'.

If you would like to make changes to your financial disclosure, please include your updated statement in your cover letter. Guidelines for resubmitting your figure files are available below the reviewer comments at the end of this letter.

If applicable, we recommend that you deposit your laboratory protocols in protocols.io to enhance the reproducibility of your results. Protocols.io assigns your protocol its own identifier (DOI) so that it can be cited independently in the future. For instructions see: https://journals.plos.org/plosone/s/submission-guidelines#loc-laboratory-protocols. Additionally, PLOS One offers an option for publishing peer-reviewed Lab Protocol articles, which describe protocols hosted on protocols.io. Read more information on sharing protocols at https://plos.org/protocols?utm_medium=editorial-email&utm_source=authorletters&utm_campaign=protocols.

As the corresponding author, your ORCID iD is verified in the submission system and will appear in the published article. PLOS supports the use of ORCID, and we encourage all coauthors to register for an ORCID iD and use it as well. Please encourage your coauthors to verify their ORCID iD within the submission system before final acceptance, as unverified ORCID iDs will not appear in the published article. *Only*  the individual author can complete the verification step; PLOS staff *cannot*  verify ORCID iDs on behalf of authors.

We look forward to receiving your revised manuscript.

Kind regards,

Gadissa Bedada Hundie, Ph.D

Academic Editor

PLOS One

Journal Requirements:

If the reviewer comments include a recommendation to cite specific previously published works, please review and evaluate these publications to determine whether they are relevant and should be cited. There is no requirement to cite these works unless the editor has indicated otherwise.

Please review your reference list to ensure that it is complete and correct. If you have cited papers that have been retracted, please include the rationale for doing so in the manuscript text, or remove these references and replace them with relevant current references. Any changes to the reference list should be mentioned in the rebuttal letter that accompanies your revised manuscript. If you need to cite a retracted article, indicate the article’s retracted status in the References list and also include a citation and full reference for the retraction notice.

Additional Editor Comments (if provided):

The reviewer has recommended acceptance and noted significant improvements in readability and methodology. Before final acceptance, please address the following minor points raised by the reviewer:

1. Supplementary Material: Consider including a supplementary figure depicting the results of your false negative rate simulation experiment, alongside the existing description in the main text.

2. Discussion Clarification: Expand slightly on the limitations and trade-offs associated with your sequence subsampling strategy (specifically, maximizing sequence divergence versus capturing recent reassortment events within clades). Ensure that lines 514–522 explicitly reflect these trade-offs as suggested by the reviewer.

[Note: HTML markup is below. Please do not edit.]

Reviewers' comments:

Reviewer's Responses to Questions

**Comments to the Author**

Reviewer #1: (No Response)

**********

2. Is the manuscript technically sound, and do the data support the conclusions?

Reviewer #1: Yes

**********

3. Has the statistical analysis been performed appropriately and rigorously?

Reviewer #1: Yes

**********

4. Have the authors made all data underlying the findings in their manuscript fully available?

The PLOS Data policy

Reviewer #1: Yes

**********

5. Is the manuscript presented in an intelligible fashion and written in standard English?

Reviewer #1: Yes

**********

Reviewer #1: The authors addressed a number of the reviewer and editor comments and made several improvements to the manuscript. Most notably, the expanded discussion and clarification of the methods in several places improves the readability of the paper significantly. The authors now include a discussion of other methods for identifying reassortment events and the ways in which those methods may or may not suffer from the same high false positive rate that SPR rearrangements do.

The authors also conducted a simulation experiment to look at the false negative rate. Perhaps these results could be included somehow in a supplementary figure, in addition to being described in the text. Importantly, by simulating reassortment in the subsampled (most divergent) control trees instead of the full trees the authors are not able to address the second major reviewer comment. The authors acknowledge this limitation in their response to the reviewers: "However, we agree that initial subsampling based solely on divergence may exclude recent rearrangement events within the clade, since these were simulated after subsampling. We have now explained this restriction in the Discussion and make it explicit that maximizing sequence divergence is appropriate for inferring general patterns of reassortment and minimizes false positive inferences due to phylogenetic reconstruction error." This explanation could be made more clearly in the Discussion. There is some relevant text (lines 514-522), but I think that it is worth addressing directly the trade-offs that are likely - even if the authors prefer not to have direct quantitative metrics - with different subsampling strategies.

Overall, my recommendation is to accept the manuscript. I think the authors could consider adding or changing wording to clarify the above point (limitations of the false negative rate experiment), but I will leave that up to the authors and I do not feel that I need to review the manuscript again.

**********

what does this mean?). If published, this will include your full peer review and any attached files.

If you choose “no”, your identity will remain anonymous but your review may still be made public.

**Do you want your identity to be public for this peer review?**  For information about this choice, including consent withdrawal, please see our Privacy Policy

Reviewer #1: No

**********

[NOTE: If reviewer comments were submitted as an attachment file, they will be attached to this email and accessible via the submission site. Please log into your account, locate the manuscript record, and check for the action link "View Attachments". If this link does not appear, there are no attachment files.]

To ensure your figures meet our technical requirements, please review our figure guidelines: https://journals.plos.org/plosone/s/figures

You may also use PLOS’s free figure tool, NAAS, to help you prepare publication quality figures: https://journals.plos.org/plosone/s/figures#loc-tools-for-figure-preparation.

NAAS will assess whether your figures meet our technical requirements by comparing each figure against our figure specifications.

# Author comment: Author response to Decision Letter 2

**DOI:** 10.1371/journal.pone.0360852.r004

29 Sep 2026

Dear colleagues,

We have addressed the additional comments raised during this round of review. Below we provide a detailed point-by-point response.

Reviewer 1

Comments

1. “Supplementary Material: Consider including a supplementary figure depicting the results of your false negative rate simulation experiment, alongside the existing description in the main text.”

Response: We have added a new supplementary figure (Fig. S4) to visualize the results of the false-negative rate simulation experiment. The figure shows the distribution of event-recovery sensitivity across the 100 replicate trees containing 10 simulated SPR events. Overall, 915 of 1,000 simulated events were recovered, corresponding to an event-level sensitivity of 91.5% and a false-negative rate of 8.5%. All 10 simulated events were recovered in 77 of the 100 replicate trees. We have also cited Fig. S4 in the Results section.

2. “The authors have expanded their discussion of subsampling to maximize sequence divergence. However, I recommend that the Discussion more explicitly reflect the trade-off between reducing phylogenetic reconstruction error and potentially excluding recent reassortment events occurring within closely related clades.”

Response: We have revised the Discussion to make this trade-off more explicit. Specifically, we clarify that initial subsampling based solely on divergence may exclude recent reassortment events within the clade, since reassortment events in our false-negative analysis were simulated after subsampling.

The following text has been added to the Discussion:

“Initial subsampling based solely on divergence may exclude recent rearrangement events within the clade, since reassortment events in our false-negative analysis were simulated after subsampling. Maximizing sequence divergence is appropriate for inferring general patterns of reassortment and minimizes false-positive inferences due to phylogenetic reconstruction error. However, the benefit of using denser sampling, albeit at the expense of phylogenetic uncertainty, might be warranted in studies that focus on recent or lineage-specific reassortment.”

Sincerely,

Hugo G. Castelán-Sánchez

on behalf of all authors

Attachment Submitted filename: responses_reviwer_v2.pdf

# Editor report: Decision Letter 2

**DOI:** 10.1371/journal.pone.0360852.r005

**Authors:** Gadissa Hundie

1 Oct 2026

Assessing the impact of phylogenetic reconstruction error on genetic reassortment inference in segmented viruses

PONE-D-26-12074R2

Dear Dr. Hugo G. Castelán-Sánchez,

We’re pleased to inform you that your manuscript has been judged scientifically suitable for publication and will be formally accepted for publication once it meets all outstanding technical requirements.

Within one week, you’ll receive an e-mail detailing the required amendments. When these have been addressed, you’ll receive a formal acceptance letter, and your manuscript will be scheduled for publication.

An invoice will be generated when your article is formally accepted. Please note, if your institution has a publishing partnership with PLOS and your article meets the relevant criteria, all or part of your publication costs will be covered. Please make sure your user information is up-to-date by logging into Editorial Manager at Editorial Manager® and clicking the ‘Update My Information' link at the top of the page. For questions related to billing, please contact billing support.

If your institution or institutions have a press office, please notify them about your upcoming paper to help maximize its impact. If they’ll be preparing press materials, please inform our press team as soon as possible—no later than 48 hours after receiving the formal acceptance. Your manuscript will remain under strict press embargo until 2 pm Eastern Time on the date of publication. For more information, please contact onepress@plos.org.

Kind regards,

Gadissa Bedada Hundie, Ph.D.

Academic Editor

PLOS One

Additional Editor Comments (optional):

Reviewers' comments:

# Editor report: Acceptance letter

**DOI:** 10.1371/journal.pone.0360852.r006

**Authors:** Gadissa Hundie

PONE-D-26-12074R2

PLOS One

Dear Dr. Castelán-Sánchez,

I'm pleased to inform you that your manuscript has been deemed suitable for publication in PLOS One. Congratulations! Your manuscript is now being handed over to our production team.

At this stage, our production department will prepare your paper for publication. This includes ensuring the following:

* All references, tables, and figures are properly cited

* All relevant supporting information is included in the manuscript submission,

* There are no issues that prevent the paper from being properly typeset

You will receive further instructions from the production team, including instructions on how to review your proof when it is ready. Please keep in mind that we are working through a large volume of accepted articles, so please give us a few days to review your paper and let you know the next and final steps.

Lastly, if your institution or institutions have a press office, please let them know about your upcoming paper now to help maximize its impact. If they'll be preparing press materials, please inform our press team within the next 48 hours. Your manuscript will remain under strict press embargo until 2 pm Eastern Time on the date of publication. For more information, please contact onepress@plos.org.

You will receive an invoice from PLOS for your publication fee after your manuscript has reached the completed accept phase. If you receive an email requesting payment before acceptance or for any other service, this may be a phishing scheme. Learn how to identify phishing emails and protect your accounts at https://explore.plos.org/phishing.

If we can help with anything else, please email us at customercare@plos.org.

Thank you for submitting your work to PLOS One and supporting open access.

Kind regards,

PLOS One Editorial Office Staff

on behalf of

Dr. Gadissa Bedada Hundie

Academic Editor

PLOS One
